# The role of gut microbiota in intestinal disease: from an oxidative stress perspective

**DOI:** 10.3389/fmicb.2024.1328324

**Published:** 2024-02-14

**Authors:** Yiqi Sun, Xurui Wang, Lei Li, Chao Zhong, Yu Zhang, Xiangdong Yang, Mingyue Li, Chao Yang

**Affiliations:** ^1^Surgery of Traditional Chinese Medicine Department, Sichuan Provincial People’s Hospital, School of Medicine, University of Electronic Science and Technology of China, Chengdu, China; ^2^Department of Anorectal Surgery, Hospital of Chengdu University of Traditional Chinese Medicine, Chengdu, China; ^3^Traditional Chinese Medicine Department of Orthopaedic and Traumatic, Sichuan Provincial People’s Hospital, School of Medicine, University of Electronic Science and Technology of China, Chengdu, China; ^4^Colorectal and Anal Surgery, Chengdu Anorectal Hospital, Chengdu, China; ^5^Special Needs Outpatient Department, Hospital of Chengdu University of Traditional Chinese Medicine, Chengdu, China

**Keywords:** gut microbiota, oxidative stress, probiotics, colitis, intestinal diseases

## Abstract

Recent studies have indicated that gut microbiota-mediated oxidative stress is significantly associated with intestinal diseases such as colorectal cancer, ulcerative colitis, and Crohn’s disease. The level of reactive oxygen species (ROS) has been reported to increase when the gut microbiota is dysregulated, especially when several gut bacterial metabolites are present. Although healthy gut microbiota plays a vital role in defending against excessive oxidative stress, intestinal disease is significantly influenced by excessive ROS, and this process is controlled by gut microbiota-mediated immunological responses, DNA damage, and intestinal inflammation. In this review, we discuss the relationship between gut microbiota and intestinal disease from an oxidative stress perspective. In addition, we also provide a summary of the most recent therapeutic approaches for preventing or treating intestinal diseases by modifying gut microbiota.

## 1 Introduction

Intestinal diseases can be broadly classified into several main categories: colorectal cancer (CRC), irritable bowel syndrome (IBS) and inflammatory bowel disease (IBD), which encompasses Crohn’s disease (CD) and ulcerative colitis (UC) (Chang, 2020; Marotto et al., 2020). The association between the occurrence of intestinal diseases and the instability of the genome induced by oxidative stress has been established (Yuan et al., 2022). Oxidative stress, a characteristic feature of intestinal diseases, including both IBD and CRC, can be initiated by dysbiosis of the gut microbiota (Bourgonje et al., 2020; Li et al., 2023; Teng et al., 2023). The human body harbors an equivalent quantity of microbial cells to that of human cells, with the preponderance of these cells primarily localized within the gastrointestinal tract (Sender and Milo, 2021; Litichevskiy and Thaiss, 2022; Sassone-Corsi et al., 2022). The term “microbiota” encompasses the entirety of commensal microbial organisms, comprising bacteria, archaea, viruses, and fungi. The significance of the gut microbiome in relation to human health and disease, specifically intestinal disease, is widely acknowledged in the current literature (Zmora et al., 2019; Fan and Pedersen, 2021; Wilmes et al., 2022). The gut microbiota comprises the assemblage of microbial species present within the gastrointestinal tract, including *Bacteroides*, *Eubacterium*, *Peptococcaceae*, *Bifidobacterium*, *Escherichia coli*, *Streptococci*, *Staphylococci*, *Lactobacillus*, and *Clostridium perfringens* (Borrelli et al., 2018; Xue et al., 2020). In addition, virions are also constituents of the gut microbiota (Sarkar et al., 2021). These organisms play a crucial role in the process of food digestion, synthesis of vitamins, and modulation of immune responses (Zhang and Chen, 2019; Li et al., 2022).

Oxidative stress, characterized by an imbalance between the generation and elimination of ROS, not only occurs in the inflamed intestinal mucosa but also extends into the deeper layers of the intestinal wall (Luceri et al., 2019; Jakubczyk et al., 2020). Numerous studies have demonstrated that the gut microbiota could modulate cellular ROS concentrations (Burgueno et al., 2021; van der Post et al., 2021; Scarano et al., 2023). *Lactobacillus* and *Bifidobacterium* residing in the gastrointestinal tract possess the capacity to enzymatically convert nitrate and nitrites into nitric oxide (NO), thereby endowing the gut epithelia with a substantial reservoir of NO (Das and Ganesh, 2023). Similarly, the production of NO can be observed in *Streptococcus* and *bacillus* through the utilization of L-arginine (Luca et al., 2019). In the context of nanomolar concentrations, NO is commonly recognized as having a protective effect. At higher concentrations, it elicits deleterious effects through the production of ROS, including superoxide (O_2_-) and hydrogen peroxide (H_2_O_2_), which subsequently give rise to highly reactive hydroxyl radicals (Lundberg et al., 2008; Kapil et al., 2020). This process has been implicated in the pathogenesis of IBD and CRC (Vona et al., 2021; Pan et al., 2022). On the other hand, the reduction in ROS is facilitated by the influence of gut bacteria-generated beneficial metabolites, specifically short-chain fatty acids (SCFAs), which are considered metabolic byproducts produced by certain bacterial species (Martins et al., 2022; Dey and Ray Chaudhuri, 2023). These SCFAs can serve as an energy source for other bacterial species through a phenomenon referred to as cross-feeding (Evans et al., 2020). Furthermore, SCFAs can directly modify the cells of the host’s intestinal tract. For example, butyrate, a prominent SCFA, is widely recognized for its role as a major energy provider for colonocytes and its contribution to the restoration of intestinal epithelial cells (Hodgkinson et al., 2023). Several research groups have reached the consensus that the microbiota residing in the small intestine exhibit the capability to metabolize glycine, an indispensable amino acid crucial for the synthesis of glutathione, participating in the redox balance (Enright et al., 2018; Kastl et al., 2020).

The dysbiosis of gut microbiota has been found to be associated with the generation of ROS, which specifically interact with cysteine redox switches present in proteins (Yardeni et al., 2019; Ballard and Towarnicki, 2020; Wang et al., 2022a). This phenomenon induces modifications in immune responses, resulting in DNA impairment and provoking inflammation within the gastrointestinal tract (Ballard and Towarnicki, 2020). The precise mechanisms by which bacteria may influence the progression of disease remain incompletely understood, despite the identification of certain members of the gut microbiota that have been implicated as causative agents in intestinal disorders (Haran and McCormick, 2021; Chakaroun et al., 2023; Ortega et al., 2023). This review provides a comprehensive analysis of the association between gut microbiota and intestinal diseases. A crucial aspect of this relationship pertains to the role of gut microbiota-mediated oxidative stress in the pathogenesis of intestinal diseases. Furthermore, we place significant emphasis on the medicinal methodologies employed to modulate the gut microbiota for the purpose of managing intestinal ailments.

## 2 The crosstalk between gut microbiota and oxidative stress

There has been a demonstrated correlation between the gut microbiota and oxidative stress in both directions over the past few decades (Shandilya et al., 2022). The gut microbiota has an impact on oxidative stress by means of metabolite synthesis, regulation of antioxidant enzymes, and maintenance of gut homeostasis (Ballard and Towarnicki, 2020; Sui et al., 2020; Pan et al., 2022). In contrast, oxidative stress has the potential to influence the gut microbiota by promoting dysbiosis (Brunt et al., 2019; Fang et al., 2023). A comprehensive comprehension of the interplay between the gut microbiota and oxidative stress is of paramount importance in elucidating the role of the gut microbiota in intestinal disorders and devising strategies to enhance gut well-being and mitigate pathologies arising from oxidative stress.

Multiple studies have demonstrated that the gut microbiota has the potential to influence the body’s oxidative stress levels through various mechanisms. The gut microbiota comprises trillions of microorganisms that actively metabolize and ferment dietary components, producing various metabolites, such as SCFAs (Kim, 2021; Nakkarach et al., 2021). Acetate, propionate, and butyrate are SCFAs that are synthesized through the process of bacterial fermentation of dietary fibers within the gastrointestinal tract (Lavelle and Sokol, 2020; Nogal et al., 2021). The observed antioxidant properties of these metabolites indicate their potential to mitigate oxidative stress in both the gastric region and systemic physiology (Michaudel and Sokol, 2020). Moreover, the modulation of antioxidant enzyme production and activity by gut bacteria enables them to regulate oxidative stress within the host (Zhou et al., 2022). For example, specific strains of bacteria, such as *Bifidobacterium longum* CCFM752, *Lactobacillus plantarum* CCFM10, and *L. plantarum* CCFM1149 can induce the production of crucial antioxidant defense enzymes such as glutathione peroxidase (Guo et al., 2022), catalase (Chen et al., 2021), and superoxide dismutase (SOD) (Wang et al., 2020). The normal gut microbiota can enhance the body’s ability to scavenge ROS and maintain redox homeostasis by promoting the synthesis of these enzymes (Yardeni et al., 2019; Zhang Y. et al., 2022). A balanced gut microbiota is essential for maintaining a healthy gut environment. The initiation and sustenance of microbial variety in the gastrointestinal tract have been associated with several factors, including early exposure to microorganisms, dietary patterns, age, geographical location, and exposure to antibiotics (Yatsunenko et al., 2012; Fassarella et al., 2021; Querdasi et al., 2023). The unhealthy conditions of the gut microbiota are identified by substantial changes in the composition and/or functionality of the microbiome, leading to a notable reduction in variety (Tran et al., 2019; Ghosh et al., 2020). This reduction may result in a decline in beneficial bacteria associated with human health, such as commensal *E. coli* (Sassone-Corsi et al., 2016), while simultaneously promoting the growth of harmful microorganisms, including *Salmonella enterica serovar Typhimurium* and *Proteobacteria* (Rivera-Chavez et al., 2016; Litvak et al., 2017). When the gut microbial balance is disrupted, deregulated bacteria can produce more ROS and impair gut barrier function (Jackson and Theiss, 2020). This disruption allows harmful substances and antigens to pass through the gut lining, further triggering oxidative stress (Singh et al., 2023).

The potential contribution of this imbalance to the development of oxidative stress-related diseases and chronic inflammation has been demonstrated. In addition, the gut microbiota can exhibit a reciprocal response to oxidative stress (Mitrea et al., 2022). Excessive generation of ROS can lead to dysbiosis, a condition characterized by an imbalance in the structure and functioning of the gut microbiota. Excessive ROS levels have been shown to cause dysbiosis of the gut microbiota by changing microbial composition, damaging epithelial barrier function and disrupting metabolic pathways (Li T. et al., 2021; Mossad et al., 2022; Collins et al., 2023). The cellular presence of ROS can exert diverse effects, encompassing the stimulation of cellular proliferation and differentiation, the release of cytokines, the induction of apoptosis-mediated cell death, and the regulation of the innate immune response (Koren and Fuchs, 2021; Morana et al., 2022). Oxidative stress can affect the balance of gut microbiota (Martens et al., 2018). For instance, it has been shown that oxidative stress may favor the growth of certain pathogenic or proinflammatory bacteria while reducing the populations of beneficial or commensal bacteria (Morais et al., 2021; Mitra et al., 2022). Excessive ROS could inflict direct damage upon the DNA and membrane structures of bacteria, resulting in cell death or a diminished ability to establish and thrive within the gastrointestinal tract (Honda and Kubes, 2018). Oxidative stress can also compromise the integrity of the gut epithelial barrier, which plays a crucial role in maintaining gut health by preventing harmful substances from entering the bloodstream (Chelakkot et al., 2018). For instance, alcohol-induced oxidative stress can disrupt the gut barrier and promote gut dysbiosis (Chen et al., 2015). Disruption of the barrier can lead to increased permeability, allowing potentially harmful bacteria and their products to cross into the bloodstream and trigger inflammation (Parker et al., 2020; Rogers et al., 2023). Furthermore, the gut microbiota plays a significant role in host metabolism, and oxidative stress can influence this interaction (Li T. et al., 2021). Dysbiosis resulting from oxidative stress might disrupt metabolic pathways in the gut, potentially leading to conditions such as obesity, diabetes, and metabolic syndrome (Tilg et al., 2020). SCFAs are metabolites produced by certain gut bacteria during the fermentation of dietary fibers. These compounds have anti-inflammatory and protective effects on the gut barrier. Oxidative stress might affect the production of SCFAs by altering the activity of bacteria responsible for their synthesis (Macia et al., 2015; Zhao et al., 2018).

**Figure 1:**
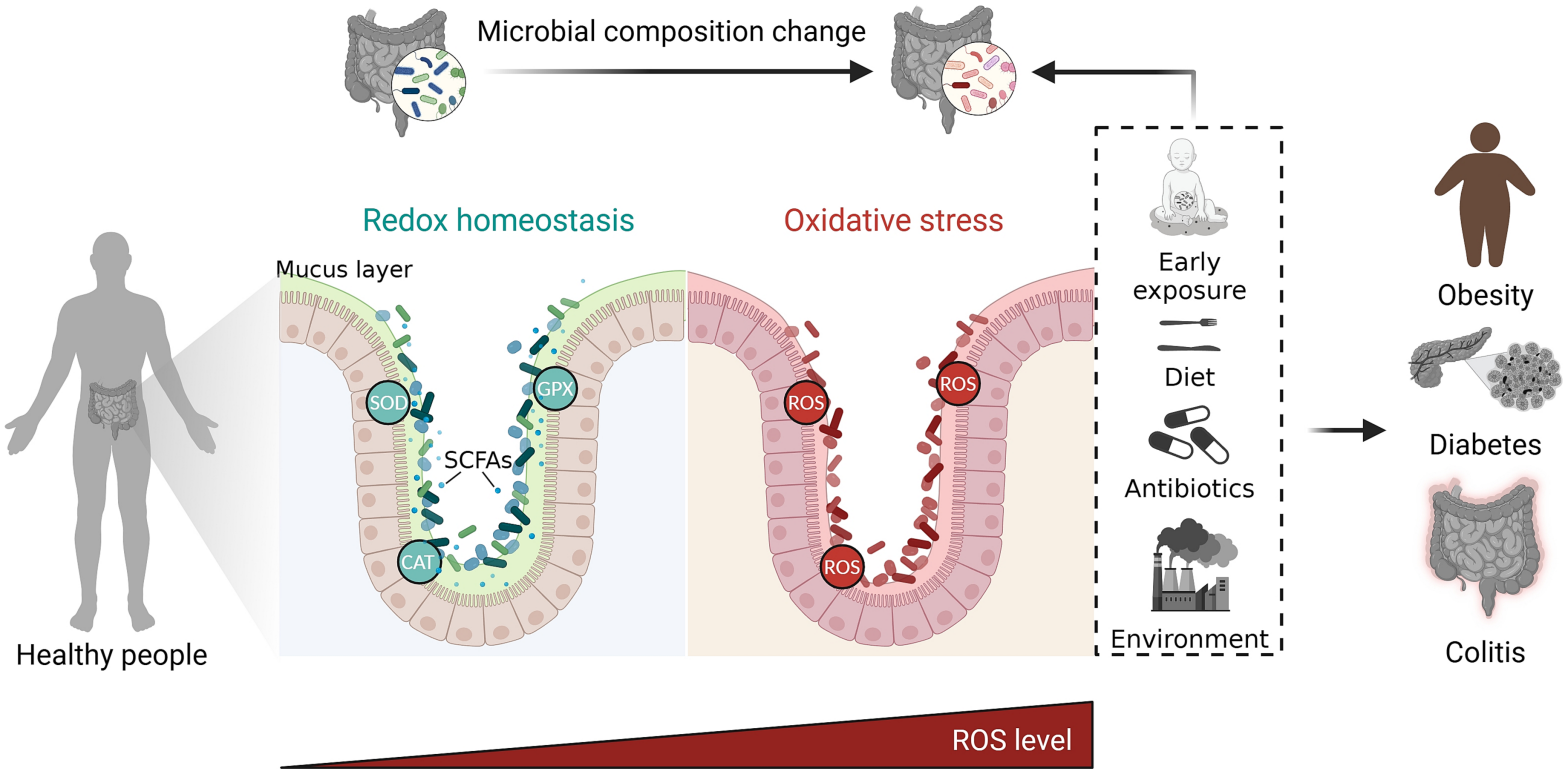
The complex and bidirectional relationship between oxidative stress and gut microbiota. The gut microbiota exerts influence on oxidative stress through regulating the production of metabolite and antioxidant enzymes. On the other hand, oxidative stress has an impact on the gut microbiota by promoting dysbiosis. Maintaining a balanced gut microbiota through a healthy diet, regular exercise, and other lifestyle factors can help mitigate the harmful effects of excessive ROS on gut health. ROS, reactive oxygen species; SOD, superoxide dismutase; CAT, catalase; GPX, gluta-thione peroxidase; SCFAs, short chain fatty acids. The figure was created with BioRender.com.

Overall, it is important to note that the relationship between oxidative stress and the gut microbiota is complex and bidirectional. While oxidative stress can influence the gut microbiota, the gut microbiota also has the capacity to impact oxidative stress levels in the body. Maintaining a balanced and diverse gut microbiota through a healthy diet, regular exercise, and other lifestyle factors can help mitigate the effects of oxidative stress on gut health. Additionally, antioxidant-rich foods and supplements may also contribute to reducing oxidative stress and supporting gut health (Figure 1).

## 3 Gut microbiota-mediated oxidative stress and bowel disease

The composition of the gut microbiota has been observed to influence the responsiveness of CRC to treatment (Gopalakrishnan et al., 2018; Wong and Yu, 2019). Additionally, a mounting body of evidence indicates a close association between the gut microbiota and the initiation and progression of various intestinal disorders (Imhann et al., 2018; Khan et al., 2019; Vona et al., 2021; Underhill and Braun, 2022; Chen X. et al., 2023). NADPH oxidase gene inactivating mutations diminish ROS generation in Crohn’s disease (Denson et al., 2018). Conversely, ulcerative colitis is linked to increased ROS generation due to elevated oxidases or mitochondrial activity (Aviello and Knaus, 2018). Maintaining a redox balance is crucial for preserving gut homeostasis, as an abnormal composition of gut microbiota has the potential to induce intestinal diseases (Aviello and Knaus, 2017). The persistent existence of ROS within the intestinal environment is a key factor in the progression of disease, as it plays a role in the initiation of chronic inflammation, immune responses and DNA damage (Wang L. et al., 2022).

### 3.1 Microbiota-mediated oxidative stress in intestinal inflammation

Extensive research has yielded compelling evidence supporting the notion that the gut microbiota plays a central role in inducing inflammation within the intestinal tract (Jackson and Theiss, 2020; Li B. et al., 2021; Chen X. et al., 2023). For instance, it has been reported that gut microorganisms including *Lactobacillus reuteri* and OTU0002 strain, a newly isolated bacterium of the Erysipelotrichaceae family, can act together to exacerbate inflammation (Miyauchi et al., 2020). Chronic inflammation begins as a defense against tissue imbalance. Due to its extended existence, it is engaged in numerous intestinal disease stages (Goguyer-Deschaumes et al., 2022). It has been demonstrated that the presence of a persistent inflammatory state in tissues is associated with an elevated risk of developing various cancers, including CRC (Ananthakrishnan et al., 2014). There exists a potential association between gut microbiota-induced inflammation and the subsequent modulation of innate and adaptive immune responses, which may be implicated in the initiation, progression, and advancement of intestinal disorders such as IBD and CRC (Ananthakrishnan et al., 2016; Cai J. et al., 2022). One illustrative instance involves segmented filamentous bacteria, which are believed to share the closest evolutionary relationship with type I Clostridia in vertebrates (Aguilera et al., 2015). These bacteria influence the activation of T helper 17 cells in the intestinal tract, thereby fostering an inflammatory milieu (Lecuyer et al., 2014). The association between cytoplasmic pattern recognition receptor nucleotide-binding oligomerization domain 2 (NOD2) and the pathogenesis of intestinal diseases has been established (Watanabe et al., 2006; Schwerd et al., 2017; Ferrand et al., 2019; De Salvo et al., 2021). Animal studies have shown a correlation between the absence of NOD2 and the occurrence of CRC (Gao et al., 2023). Garo et al. found that miR-146a binds Receptor-interacting protein kinase 2 (RIPK2), a NOD2 signaling intermediary, to inhibit myeloid cell-derived IL-17-inducing cytokines and colonic IL-17, inhibiting colonic inflammation and carcinogenesis (Garo et al., 2021). The presence of an abnormal microbiota, resulting from a deficiency in NOD2, has been observed to facilitate inflammation and the development of cancer (Zhou et al., 2021). Furthermore, it has been demonstrated that intestinal diseases can be transmitted to healthy mice by means of gut microorganisms, and the existence of an altered microbiota in patients with NOD2 deficiency has been found to promote the process (Couturier-Maillard et al., 2013).

Lipopolysaccharide (LPS), also known as endotoxin, is an additional inflammatory mediator (Muendlein et al., 2022; Park et al., 2023). Gram-negative bacteria, predominantly located in the gastrointestinal tract and oral cavity, contain this particular constituent (Simpson and Trent, 2019; Stephens and von der Weid, 2020). Recent studies have provided evidence indicating that LPS could initiate the TLR4 pathway, leading to the development of endothelial dysfunction and vascular inflammation (Wu et al., 2022). The activation of this pathway occurs in response to the initiation of vascular oxidative stress by LPS (Wang Y. T. et al., 2022; Yoon et al., 2022).

### 3.2 Microbiota-mediated oxidative stress in immune responses

The microbial composition of the gastrointestinal tract exerts a substantial influence on the maintenance of immune system equilibrium within the intestinal region (Erttmann et al., 2022). One of the primary functions of the gut microbiota is to safeguard the body against colonization by pathogens and the proliferation of indigenous pathobionts, which may arise due to disruptions in the equilibrium of the healthy microbial community (Shi et al., 2017; Kitamoto et al., 2020). The mechanisms underlying the ability of the microbiota to suppress the proliferation of pathogenic microorganisms are intricate. The mechanisms encompass competitive metabolic interactions, the localization of microbes within specific intestinal niches, and the elicitation of immunological responses by the host (Fung et al., 2019; Osbelt et al., 2021; Lyu et al., 2022). The phenomenon of oxidative stress possesses the capacity to exert substantial effects on immune cells, encompassing their activation and regulation (Datta et al., 2017).

The gut microbiota can exert various effects on oxidative stress. Gut *Lactobacillus*, *Bifidobacterium*, *Salmonella*, *E. coli* and *Streptococcus* are involved in the production of ROS. In addition, *B. longum* CCFM752, *L. plantarum* CCFM10, and *L. plantarum* CCFM1149 are reported to play a role in neutralization of ROS by upregulating the production of antioxidant enzymes. Moreover, it is important to note that dysbiosis can induce a disruption in the integrity of the gastrointestinal barrier (Chopyk and Grakoui, 2020; Bai et al., 2022). This disruption can subsequently result in an elevation of intestinal permeability, allowing microbial components to enter the systemic circulation (Cox et al., 2022). Microbial compounds, such as LPS, possess the capacity to stimulate immune cells, thereby inducing oxidative stress and facilitating the production of ROS (Grosheva et al., 2020; Zhang B. et al., 2022).

Previous studies have provided evidence to support the notion that the immune system’s responses can be affected by oxidative stress, which is modulated by the microbial community residing in the gastrointestinal tract (Grosheva et al., 2020; Liu et al., 2021; Metta et al., 2022). It has been demonstrated that the composition of the gastrointestinal microbiota can influence antitumor immune responses and modulate the efficacy of cancer immunotherapies, specifically immune checkpoint inhibitors (ICIs) (McCulloch et al., 2022; Mirji et al., 2022). Metabolites play a crucial role in modulating antitumor immunity through their interactions with the gut microbiota (Wang and Zhao, 2018). Metabolites are small molecules that can migrate from their original location within the gastrointestinal tract to various regions of the body (Jabot et al., 2016). Once disseminated, these metabolites can influence both the localized and systemic antitumor immune response, thereby augmenting the efficacy of ICIs (Hayase and Jenq, 2021). Moreover, empirical evidence suggests that the gut microbiota may contribute to the body’s defense against the deleterious consequences of oxidative stress (Mazenc et al., 2022). Gut commensals produce significant quantities of hydrogen sulfide (H_2_S), which subsequently undergoes conversion by the epithelium into thiosulfate (S_2_O_3_^2−^) (Walsh et al., 2022). This conversion process serves to safeguard host cells against the detrimental effects associated with H_2_S (Ran et al., 2022). The presence of Salmonella infection triggers the recruitment of neutrophils, which subsequently induces the generation of ROS (Schurmann et al., 2017). Consequently, the S_2_O_3_^2^-compound undergoes conversion into tetrathionate (S_4_O_6_^2−^) (Rogers et al., 2021). Salmonella, in contrast to commensal microorganisms, harbors the operon ttrSR ttrBCA, which confers the ability to metabolize S_4_O_6_^2−^ (Kamada et al., 2013; Sato et al., 2021). In an inflammatory environment, Salmonella is able to gain a growth advantage over commensal bacteria (Ali et al., 2014). Furthermore, the compound S_4_O_6_^2−^ has been observed to facilitate the growth and proliferation of Salmonella bacteria under anaerobic conditions when supplemented with ethanolamine (Goes et al., 2022).

In summary, the intricate interplay among gut microbiota, oxidative stress, and immunological responses exerts a substantial influence on the pathogenesis of intestinal disorders (Jones and Neish, 2021; de Vos et al., 2022). Various factors can contribute to the onset of these diseases. Dysbiosis-induced oxidative stress disrupts the intricate equilibrium of gut homeostasis, leading to an increased degree of immunological dysregulation (Pellon et al., 2021). The identification of fresh therapeutic targets for the treatment and prevention of intestinal illnesses may be possible if a comprehensive understanding of the underlying processes governing this interaction is achieved. In subsequent investigations, it is recommended that scholarly focus be directed toward the elucidation of intricate molecular pathways implicated in the regulation of gut microbiota and redox equilibrium. Furthermore, diverse methodologies should be explored to effectively manipulate these pathways, with the ultimate objective of reinstating intestinal homeostasis and impeding the progression of pathological conditions.

### 3.3 Microbiota-mediated oxidative stress in DNA damage

The occurrence of DNA damage plays a pivotal role in the advancement of various gastrointestinal conditions, such as IBD and CRC (Pellon et al., 2021). The etiology of this condition encompasses a diverse range of factors, such as oxidative stress, genotoxic agents, and infectious pathogens, among other etiological contributors (Bednarski and Sleckman, 2019; Hopfner and Hornung, 2020; Xu et al., 2023). The dysbiotic gut microbiota can generate an excessive amount of ROS, which can directly target DNA and cause various forms of damage, including single-strand breaks, double-strand breaks, and modifications to DNA base sequences (Li Q. et al., 2021; Barnes et al., 2022; Ray et al., 2022; Wang et al., 2022b). The accumulation of DNA damage may be further intensified due to the potential impact of oxidative stress on the efficiency of DNA repair mechanisms (Srivastava et al., 2020). commensal bacteria-induced oxidative stress that results in age-dependent decline in DNA damage repair and Germ-free mice showed improvement in age-related DNA damage. There exists a potential association between the occurrence of CRC and specific constituents of the gut microbiota, owing to their capacity to produce a substantial quantity of genotoxic substances (Janney et al., 2020; Cao et al., 2022). As an illustration, it has been determined that *Enterococcus faecalis* is capable of generating hydroxyl radicals (·OH) (Huycke and Moore, 2002; Bianco et al., 2017). The gram-positive bacterium under consideration is accountable for the production of substantial quantities of extracellular oxygen, resulting in the formation of hydrogen peroxide (H_2_O_2_) and ·OH (Caianelo et al., 2017). In instances of colorectal adenocarcinoma (CAC), alterations affecting the Wnt/β-catenin signaling pathway manifest exclusively during advanced disease stages, subsequent to the occurrence of mutations in the TP53 and K-Ras genes (Intarajak et al., 2019). The accumulation of DNA damage over a period of time can have significant implications for cellular function and play a role in the development and advancement of intestinal disorders (Hu et al., 2016). These effects can arise due to various factors. The gut microbiota is responsible for the production of a wide array of compounds, each possessing the capacity to induce DNA damage either through direct or indirect mechanisms (Pleguezuelos-Manzano et al., 2020). For example, certain bacterial metabolites, including secondary bile acids, possess the capacity to induce genotoxicity and inflict harm upon DNA (Cai Y. et al., 2022). Furthermore, previous studies have provided evidence indicating that SCFAs possess the ability to alter the functionality of enzymes responsible for DNA repair (Singh et al., 2018; Lipska et al., 2020). This suggests that SCFAs have the potential to impact the cellular response to DNA damage. In addition, colibactin, the canonical microbiota-derived genotoxin produced by commensal *E. coli* strains, and small-molecule genotoxins indolimines produced by multiple *Morganella morganii* strains have been reported to directly damage DNA (Cao et al., 2022).

**Figure 2:**
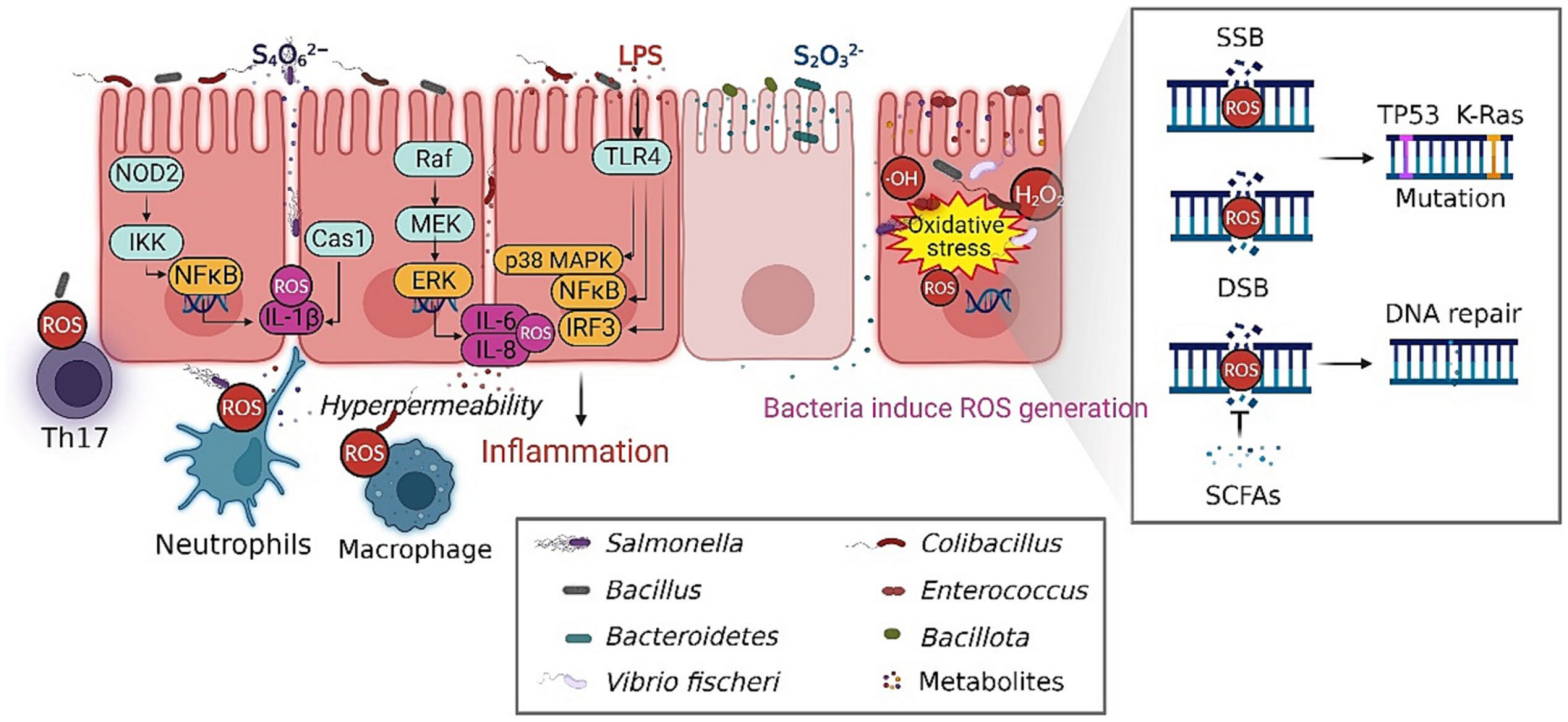
The composition of the gut microbiota influence the initiation and progression of intestinal diseases. The persistent existence of ROS within the intestinal environment is a key factor in the progression of disease, which plays an essential role in the initiation of chronic inflammation, immune responses and DNA damage. The gut-residing microbiota can generate metabolites endowed with antioxidant properties, thereby mitigating the extent of DNA damage resulting from oxidative stress. The dysbiotic gut microbiota significantly disrupt the intricate equilibrium between the generation of ROS and the process of detoxification, contributing to the development of intestinal diseases. SSB, single strand break; DSB double strand breaks. The figure was created with BioRender.com.

Conversely, the gut-residing microbiota can generate metabolites endowed with antioxidant properties (Lin et al., 2022). The metabolites possess the ability to effectively scavenge ROS and thereby mitigate the extent of DNA damage resulting from oxidative stress (Woodby et al., 2020). The aforementioned discoveries offer valuable understanding regarding the complex interplay between gut microbiota, microbial metabolites, and DNA damage (Puschhof and Sears, 2022). In general, the dysbiotic gut microbiota significantly disrupts the intricate equilibrium between the generation of ROS and the process of detoxification, thereby contributing to both DNA damage and the development of intestinal diseases (Figure 2).

## 4 Gut microbiota-modulating therapeutic approaches for bowel disease

**Figure 3:**
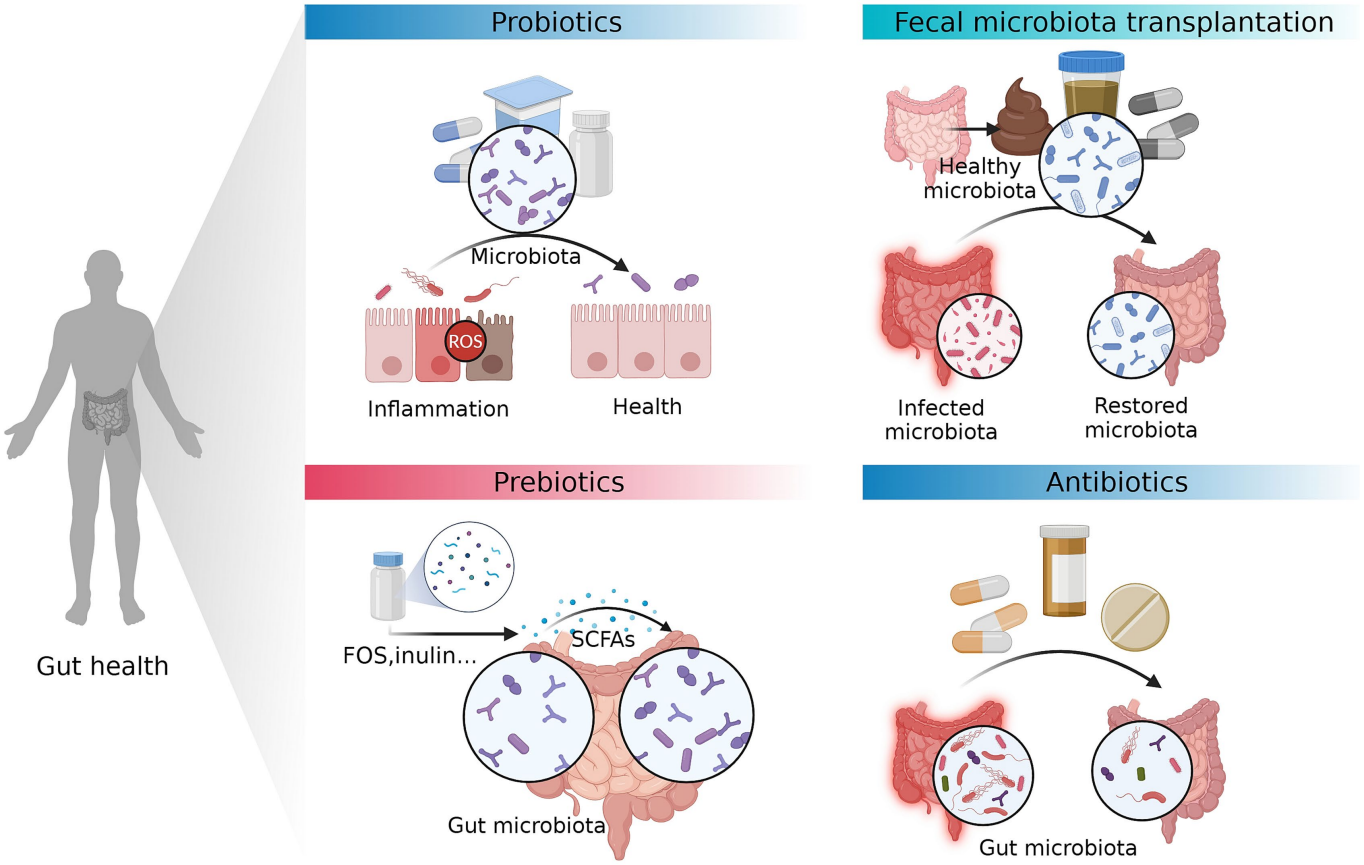
Therapeutic approaches that can prevent or treat intestinal diseases by modifying gut microbiota. The manipulation of the gut microbiota composition via prebiotics, probiotics, fecal microbiota transplantation (FMT), and antibiotics exhibits potential as a therapeutic approach for reestablishing redox equilibrium and mitigating immunological dysregulation. FOS, fructo-oligosaccharides. The figure was created with BioRender.com.

Gut dysbiosis is a prevalent characteristic in the pathophysiology of various intestinal disorders. Recent research has generated substantial evidence illustrating the protective role of the gut microbiota in the human body (Schuijt et al., 2016; Wieczorska et al., 2020). Various factors, including variable variables such as food and medications, as well as host factors such as age and genetics, have the potential to induce alterations in the composition of gut microbiota (Schupack et al., 2022). Moreover, these factors can also elicit changes in the signaling activity of gut microbiota (Cao et al., 2023). The hypothesis posits that manipulating the human gut, which is involved in a wide range of physiological processes, may have the potential to prevent or treat disorders linked to these functions (Tripathi et al., 2018; Bajaj et al., 2019; Shin et al., 2019). Hence, the manipulation of the gut microbiota composition via prebiotics, probiotics, fecal microbiota transplantation (FMT), and antibiotics exhibits potential as a therapeutic approach for reestablishing redox equilibrium and mitigating immunological dysregulation (Figure 3).

### 4.1 Probiotics

Probiotics refer to living microorganisms that, when consumed in adequate amounts, can confer advantageous effects on an individual’s well-being (Hill et al., 2014; Mitrea et al., 2023). *Saccharomyces cerevisiae boulardii*, the gram-negative *E. coli* strain Nissle 1917, a number of lactic-acid-producing lactobacilli strains, and various bifidobacterial strains are the primary microorganisms classified as probiotic agents (Hill et al., 2014). These beneficial microorganisms possess the capacity to reinstate the equilibrium of microorganisms in the gastrointestinal tract, enhance the functionality of the intestinal barrier, and modify immunological reactions (Ziemons et al., 2021). Several studies have been conducted to investigate the potential impact of probiotics on the management of gastrointestinal disorders, including IBS, IBD, necrotizing enterocolitis, and diarrhea (Goldenberg et al., 2017; Gracie et al., 2019; Mei et al., 2022; Nabavi-Rad et al., 2022). The utility of probiotics extends beyond their nutritional value, as they play a crucial role in enhancing and fortifying the existing gut microbiota (Darwish et al., 2022). Indeed, it is possible for them to modify the composition of the indigenous gut microbiota, resulting in a subsequent modification of the gut lumen that promotes a more advantageous anti-inflammatory environment (Suez et al., 2018). As a consequence, there is a decrease in the production of bacterial products that promote inflammation and an enhancement in the integrity of the gastrointestinal barrier (Leta et al., 2021). It has been demonstrated that certain strains of probiotics, namely, those belonging to the *Lactobacillus* and *Bifidobacterium* species, can diminish disease activity, mitigate symptoms, and enhance overall quality of life (Ashraf and Shah, 2014). For instance, previous studies have provided evidence that the administration of *Lactobacillus rhamnosus* GG could potentially restore the balance of dysbiotic microbiota, leading to various beneficial effects on intestinal functionality (Moens et al., 2019). Furthermore, it has been reported that LGG possesses the ability to mitigate oxidative stress within the gastrointestinal tract (Hou et al., 2019; Li J. et al., 2021; Ma K. et al., 2022). Daily consumption of LGG affected intestinal oxidative stress and the severity of steatohepatitis in a rat model that simulated alcohol-induced leaky gut (Wang et al., 2011). Researchers demonstrated that regular administration of LGG therapy resulted in the restoration of intestinal barrier function, reduction in markers of oxidative stress and inflammation in the intestines, and significant mitigation of the severity of alcohol-induced intestinal permeability (Wang et al., 2011; Chen L. et al., 2023).

There is potential for probiotic-derived compounds, such as surface-layer proteins and bacteriocins, to exhibit advantageous effects beyond their interaction with living organisms (Kirk et al., 2017; Butorac et al., 2020; Chandla et al., 2021). For instance, pretreatment with *Bifidobacterium longum* subspecies *infantis* conditioned medium (BiCM) prevented the inflammatory cytokines IFN-γ and TNF-α induced ROS damage and the drop in transepithelial electrical resistance (TER), suggesting that this particular organism is capable of secreting a bioactive substance (Lomasney et al., 2014). Furthermore, the utilization of probiotic DNA therapy derived from the probiotic combination VSL#3 probiotic mixture has demonstrated the capacity to decrease both the overall disease activity and inflammation in the colon of IL-10^−/−^ animals (Cruz et al., 2020).

### 4.2 Prebiotics

Prebiotics refer to a category of dietary fiber that, despite being indigestible, possesses the capacity to selectively enhance the growth and functionality of advantageous bacteria within the gastrointestinal tract (Sanders et al., 2019). For instance, silver fir (*Abies alba*) bark extract showed antioxidant activity and acted as a prebiotic for *Lactobacillus* species bacteria including: *L. paracasei*, *L. acidophilus*, *L. rhamnosus*, *L. gasseri*, *L. crispatus* and *L. bulgaricus* (Stojanov et al., 2021). Prebiotics function by serving as a substrate for the growth of specific microorganisms, thereby stimulating the production of SCFAs and other compounds with anti-inflammatory properties (Dalile et al., 2019; Pujo et al., 2021). These substances play a crucial role in the regulation of immunological responses, the preservation of gut integrity, and the reinstatement of microbial diversity (Quigley, 2019). The utilization of prebiotic supplements has exhibited potential as a strategy for alleviating the symptoms of gastrointestinal disorders and improving their clinical outcomes (Ford et al., 2018). Considerable research has been dedicated to investigating the impact of inulin, a type of fructan-based prebiotic, on the gut microbiota (Nicolucci et al., 2017). Clinical studies have demonstrated that the administration of an inulin supplement in individuals diagnosed with IBD and IBS promotes the proliferation of *Bifidobacterium* and *Lactobacillus* species (Vandeputte et al., 2017). This microbial modulation leads to a favorable shift in the balance of beneficial and pathogenic bacteria, thereby enhancing the overall composition of the gut microbiota. Previous studies have provided evidence to support the notion that fructo-oligosaccharides (FOS), a type of prebiotic, have the potential to effectively regulate the composition of gut microbiota (Burokas et al., 2017). It has been demonstrated that the consumption of FOS supplements leads to an increase in the abundance of beneficial bacteria associated with gastrointestinal well-being, such as *Bifidobacterium* and *Faecalibacterium prausnitzii* (Chi et al., 2020). Galacto-oligosaccharides (GOS) are another type of prebiotic derived from lactose and are known to exert beneficial effects on the gut microbiota (Arnold et al., 2021). After the administration of GOS, clinical studies have observed an increase in the populations of *Bifidobacterium* and *Lactobacillus* (Monteagudo-Mera et al., 2016). The variability of individual responses to prebiotics underscores the necessity for personalized strategies and a more comprehensive comprehension of the interplay between the gut microbiota and the host (Bedu-Ferrari et al., 2022). While there is evidence linking modulation of the gut microbiota to decreased disease activity and improved gastrointestinal symptoms in individuals with intestinal diseases, it is important to note that the response to prebiotics can vary among individuals.

### 4.3 Fecal microbiota transplantation

The utilization of fecal microbiota transplantation (FMT) is experiencing a significant surge in recognition as a therapeutic intervention with the potential to influence the progression of various chronic conditions, particularly those affecting the gastrointestinal system (Gupta and Khanna, 2017; Costello et al., 2019). Furthermore, it has contributed to the elucidation of the function of the gut microbiome (Ooijevaar et al., 2019). Although FMT techniques have yielded valuable mechanistic insights, the practical implementation of these techniques in clinical settings may encounter limitations arising from various challenges associated with metabolic disorders (Parker et al., 2022). In certain cases, the afore mentioned phenomenon may lead to the development of recurrent pseudomembranous colitis, a condition characterized by the excessive proliferation of the bacterium *Clostridioides difficile* infection. It is hypothesized that the transplantation of fecal microbiota from a healthy donor can effectively restore the gut microbiome and prevent the further dissemination of *C. difficile* infection (Guery et al., 2019). It is challenging, subsequent to the culmination of several clinical trials that have illustrated the effectiveness of FMT as a therapeutic intervention for this particular ailment. The utilization of FMT has been explored in the context of various chronic conditions, such as gastrointestinal disorders and ulcerative colitis, alongside various other medical conditions (Costello et al., 2019). It is important to note that the reliability and validity of the findings obtained from these investigations are subject to significant variation, which can be attributed to factors such as the study’s methodology and the sample size of participants. It is worth noting that fecal transplants encompass more than solely the microbiota or microbiome (Dsouza et al., 2022). This issue is considered to be of utmost importance within the context of FMT. Moreover, clinical trials involving FMT are often conducted on patient subgroups who exhibit a high degree of resistance to conventional treatments, such as individuals afflicted with refractory CD (Langdon et al., 2021). FMT has emerged as a pivotal approach in examining the causal role of the microbiome in various chronic diseases. Despite its inherent limitations, it remains a significant tool in this field. To progress beyond its utilization as a final option treatment in clinical settings for recurrent *C. difficile*, it is imperative to prioritize the implementation of additional measures to standardize FMT procedures.

### 4.4 Antibiotics

Antibiotics are commonly employed as a conventional therapeutic approach for the management of infectious diarrhea, a condition that can arise due to diverse bacterial infections, such as *Salmonella*, *Shigella*, and *Campylobacter* (Maier et al., 2021). Antimicrobial agents that specifically target pathogenic microorganisms associated with the illness have the capacity to eliminate these microorganisms, thereby alleviating symptoms and mitigating potential complications (McDonnell et al., 2021). Antibiotic kill bacteria in part by inducing oxidative damage. For instance, streptomycin can stimulate the Fenton reaction and effectively contribute to cell killing in *E. coli*, which could be prevented by overexpressing a ROS scavenger (Lv et al., 2023). Antibiotics are commonly employed for the purpose of eradicating pathogenic bacteria; however, they also possess the capacity to modify the composition of the gut microbiota (Ma P. et al., 2022). Antibiotics may alleviate dysbiosis by inhibiting the proliferation of pathogenic bacteria that are associated with the progression of gastrointestinal disorders (Yin et al., 2018). The administration of antibiotics such as metronidazole, ciprofloxacin, and rifaximin has been demonstrated to effectively reduce disease exacerbations and modulate the composition of the intestinal microbiota in the management of IBD and CD (Limketkai et al., 2020; Strati et al., 2021). These antibiotics could modify the composition and diversity of the gut microbiota, potentially leading to a reduction in the occurrence of certain harmful species and promoting a more advantageous microbial balance (Stevens et al., 2022). Nevertheless, the prolonged and indiscriminate use of antibiotics may disrupt the intricate microbial equilibrium within the gastrointestinal tract, potentially leading to antibiotic-associated diarrhea and the emergence of drug-resistant bacterial strains (Schwartz et al., 2020). Therefore, customized approaches are necessary in the selection of antibiotics for the management of bowel diseases owing to variations in the gut microbiota and disease characteristics among individuals. To optimize treatment outcomes, it is imperative to carefully consider the potential benefits of antibiotics alongside the risks associated with dysbiosis, harm to commensal bacteria, and long-term effects.

## 5 Conclusion and perspective

The microbial population residing in the gastrointestinal tract is approximately comparable in size to the population of human cells distributed throughout the entirety of the body. The gut microbiota possesses the capacity to perceive, regulate, and disseminate an extensive array of chemical signals originating from the surrounding milieu. This phenomenon significantly affects the well-being of individuals. The regulation of redox balance in the intestinal tract is influenced by the presence of gut bacteria, either through direct or indirect mechanisms. A microbiome in a state of equilibrium exhibits a propensity for maintaining redox balance, whereas dysbiosis disrupts this state of equilibrium. The aggregation of preclinical evidence has indicated that the manipulation of gut microbiota holds promise as a therapeutic approach for the prevention and treatment of intestinal diseases. This review discusses the correlation between gut microbiota and oxidative stress, as well as the potential role of gut bacteria in modulating the susceptibility of the intestine to oxidative stress. The extent to which the gut microbiota contributes to intestinal diseases can be inferred to a limited degree by examining the presence or absence of specific bacterial species. The alteration of viromes and fungal microbiota has been associated with CRC, implying a potential interaction between these microorganisms and gut bacteria that could potentially influence patients’ responses to cancer therapy. The investigation of the causal connections between bacteria and intestinal disorders, as well as the underlying mechanisms involved, has been the focus of extensive scholarly research. The gut microbiota plays a crucial role in the synthesis of various metabolites, which subsequently influence numerous biological processes, including the modulation of the immune system. In the future, it is anticipated that significant advancements will be made in the field of medical research, particularly in relation to the discovery of bacteria-derived metabolites and enzymes that may contribute to the onset or progression of intestinal disorders. These forthcoming findings are expected to exert a substantial influence on the approach employed for the treatment of such ailments. Furthermore, due to the significant role of oxidative stress in the pathogenesis of intestinal disorders, the manipulation of gut microbiota through the administration of antioxidants can be considered a potential strategy for the prevention of intestinal diseases.

## Author contributions

YS: Data curation, Writing – original draft. XW: Data curation, Writing – original draft. LL: Data curation, Writing – original draft. CZ: Visualization, Writing – review & editing. YZ: Visualization, Writing – review & editing. XY: Writing – review & editing. ML: Conceptualization, Funding acquisition, Supervision, Writing – review & editing. CY: Conceptualization, Funding acquisition, Supervision, Writing – review & editing.

## References

[ref1] Aguilera M. Cerda-Cuellar M. Martinez V. (2015). Antibiotic-induced dysbiosis alters host-bacterial interactions and leads to colonic sensory and motor changes in mice. Gut Microbes 6, 10–23. doi: 10.4161/19490976.2014.990790, PMID: 25531553 PMC4615720

[ref2] Ali M. M. Newsom D. L. Gonzalez J. F. Sabag-Daigle A. Stahl C. Steidley B.. (2014). Fructose-asparagine is a primary nutrient during growth of Salmonella in the inflamed intestine. PLoS Pathog. 10:e1004209. doi: 10.1371/journal.ppat.1004209, PMID: 24967579 PMC4072780

[ref3] Ananthakrishnan A. N. Cagan A. Cai T. Gainer V. S. Shaw S. Y. Churchill S.. (2016). Statin use is associated with reduced risk of colorectal Cancer in patients with inflammatory bowel diseases. Clin. Gastroenterol. Hepatol. 14, 973–979. doi: 10.1016/j.cgh.2016.02.017 26905907 PMC4912917

[ref4] Ananthakrishnan A. N. Cheng S. C. Cai T. Cagan A. Gainer V. S. Szolovits P.. (2014). Serum inflammatory markers and risk of colorectal cancer in patients with inflammatory bowel diseases. Clin. Gastroenterol. Hepatol. 12, 1342–1348.e1. doi: 10.1016/j.cgh.2013.12.030, PMID: 24407106 PMC4085150

[ref5] Arnold J. W. Roach J. Fabela S. Moorfield E. Ding S. Blue E.. (2021). The pleiotropic effects of prebiotic galacto-oligosaccharides on the aging gut. Microbiome 9:31. doi: 10.1186/s40168-020-00980-0, PMID: 33509277 PMC7845053

[ref6] Ashraf R. Shah N. P. (2014). Immune system stimulation by probiotic microorganisms. Crit. Rev. Food Sci. Nutr. 54, 938–956. doi: 10.1080/10408398.2011.619671 24499072

[ref7] Aviello G. Knaus U. G. (2017). ROS in gastrointestinal inflammation: rescue or sabotage? Br. J. Pharmacol. 174, 1704–1718. doi: 10.1111/bph.13428, PMID: 26758851 PMC5446568

[ref8] Aviello G. Knaus U. G. (2018). NADPH oxidases and ROS signaling in the gastrointestinal tract. Mucosal Immunol. 11, 1011–1023. doi: 10.1038/s41385-018-0021-8, PMID: 29743611

[ref9] Bai X. Wei H. Liu W. Coker O. O. Gou H. Liu C.. (2022). Cigarette smoke promotes colorectal cancer through modulation of gut microbiota and related metabolites. Gut 71, 2439–2450. doi: 10.1136/gutjnl-2021-325021, PMID: 35387878 PMC9664112

[ref10] Bajaj J. S. Sharma A. Dudeja P. K. (2019). Collaborators targeting gut microbiome interactions in service-related gastrointestinal and liver diseases of veterans. Gastroenterology 157, 1180–1183.e1. doi: 10.1053/j.gastro.2019.07.060, PMID: 31404532 PMC7249241

[ref11] Ballard J. W. O. Towarnicki S. G. (2020). Mitochondria, the gut microbiome and ROS. Cell. Signal. 75:109737. doi: 10.1016/j.cellsig.2020.109737, PMID: 32810578

[ref12] Barnes R. P. de Rosa M. Thosar S. A. Detwiler A. C. Roginskaya V. Van Houten B.. (2022). Telomeric 8-oxo-guanine drives rapid premature senescence in the absence of telomere shortening. Nat. Struct. Mol. Biol. 29, 639–652. doi: 10.1038/s41594-022-00790-y, PMID: 35773409 PMC9287163

[ref13] Bednarski J. J. Sleckman B. P. (2019). At the intersection of DNA damage and immune responses. Nat. Rev. Immunol. 19, 231–242. doi: 10.1038/s41577-019-0135-6, PMID: 30778174 PMC6438741

[ref14] Bedu-Ferrari C. Biscarrat P. Langella P. Cherbuy C. (2022). Prebiotics and the human gut microbiota: from breakdown mechanisms to the impact on metabolic health. Nutrients 14:2096. doi: 10.3390/nu14102096, PMID: 35631237 PMC9147914

[ref15] Bianco A. Polo Lopez M. I. Fernandez Ibanez P. Brigante M. Mailhot G. (2017). Disinfection of water inoculated with *Enterococcus faecalis* using solar/Fe(III)EDDS-H_2_O_2_ or S_2_O_8_^2−^ process. Water Res. 118, 249–260. doi: 10.1016/j.watres.2017.03.061 28433695

[ref16] Borrelli A. Bonelli P. Tuccillo F. M. Goldfine I. D. Evans J. L. Buonaguro F. M.. (2018). Role of gut microbiota and oxidative stress in the progression of non-alcoholic fatty liver disease to hepatocarcinoma: current and innovative therapeutic approaches. Redox Biol. 15, 467–479. doi: 10.1016/j.redox.2018.01.009, PMID: 29413959 PMC5975181

[ref17] Bourgonje A. R. Feelisch M. Faber K. N. Pasch A. Dijkstra G. van Goor H. (2020). Oxidative stress and redox-modulating therapeutics in inflammatory bowel disease. Trends Mol. Med. 26, 1034–1046. doi: 10.1016/j.molmed.2020.06.006, PMID: 32620502

[ref18] Brunt V. E. Gioscia-Ryan R. A. Richey J. J. Zigler M. C. Cuevas L. M. Gonzalez A.. (2019). Suppression of the gut microbiome ameliorates age-related arterial dysfunction and oxidative stress in mice. J. Physiol. 597, 2361–2378. doi: 10.1113/JP277336, PMID: 30714619 PMC6487935

[ref19] Burgueno J. F. Fritsch J. Gonzalez E. E. Landau K. S. Santander A. M. Fernandez I.. (2021). Epithelial TLR4 signaling activates DUOX2 to induce microbiota-driven tumorigenesis. Gastroenterology 160, 797–808.e6. doi: 10.1053/j.gastro.2020.10.031, PMID: 33127391 PMC7879481

[ref20] Burokas A. Arboleya S. Moloney R. D. Peterson V. L. Murphy K. Clarke G.. (2017). Targeting the microbiota-gut-brain Axis: prebiotics have anxiolytic and antidepressant-like effects and reverse the impact of chronic stress in mice. Biol. Psychiatry 82, 472–487. doi: 10.1016/j.biopsych.2016.12.031, PMID: 28242013

[ref21] Butorac K. Banic M. Novak J. Lebos Pavunc A. Uroic K. Durgo K.. (2020). The functional capacity of plantaricin-producing *Lactobacillus plantarum* SF9C and S-layer-carrying *Lactobacillus brevis* SF9B to withstand gastrointestinal transit. Microb. Cell Factories 19:106. doi: 10.1186/s12934-020-01365-6, PMID: 32430020 PMC7236188

[ref22] Cai Y. Shen X. Lu L. Yan H. Huang H. Gaule P.. (2022). Bile acid distributions, sex-specificity, and prognosis in colorectal cancer. Biol. Sex Differ. 13:61. doi: 10.1186/s13293-022-00473-9, PMID: 36274154 PMC9590160

[ref23] Cai J. Sun L. Gonzalez F. J. (2022). Gut microbiota-derived bile acids in intestinal immunity, inflammation, and tumorigenesis. Cell Host Microbe 30, 289–300. doi: 10.1016/j.chom.2022.02.004, PMID: 35271802 PMC8923532

[ref24] Caianelo M. Rodrigues-Silva C. Maniero M. G. Guimaraes J. R. (2017). Antimicrobial activity against gram-positive and gram-negative bacteria during gatifloxacin degradation by hydroxyl radicals. Environ. Sci. Pollut. Res. Int. 24, 6288–6298. doi: 10.1007/s11356-016-6972-y, PMID: 27376368

[ref25] Cao Y. Liu B. Li W. Geng F. Gao X. Yue L.. (2023). Protopanaxadiol manipulates gut microbiota to promote bone marrow hematopoiesis and enhance immunity in cyclophosphamide-induced immunosuppression mice. MedComm 4:e222. doi: 10.1002/mco2.222 36845073 PMC9950037

[ref26] Cao Y. Oh J. Xue M. Huh W. J. Wang J. Gonzalez-Hernandez J. A.. (2022). Commensal microbiota from patients with inflammatory bowel disease produce genotoxic metabolites. Science 378:eabm3233. doi: 10.1126/science.abm3233, PMID: 36302024 PMC9993714

[ref27] Chakaroun R. M. Olsson L. M. Backhed F. (2023). The potential of tailoring the gut microbiome to prevent and treat cardiometabolic disease. Nat. Rev. Cardiol. 20, 217–235. doi: 10.1038/s41569-022-00771-0, PMID: 36241728

[ref28] Chandla S. Harjai K. Shukla G. (2021). Synergistic effect of Biogenics derived from potential probiotics together with Zingerone against biofilm formation by *Pseudomonas aeruginosa* PAO1. Probiotics Antimicrob Proteins 13, 1481–1497. doi: 10.1007/s12602-021-09763-x 33783720

[ref29] Chang J. T. (2020). Pathophysiology of inflammatory bowel diseases. N. Engl. J. Med. 383, 2652–2664. doi: 10.1056/NEJMra2002697 33382932

[ref30] Chelakkot C. Ghim J. Ryu S. H. (2018). Mechanisms regulating intestinal barrier integrity and its pathological implications. Exp. Mol. Med. 50, 1–9. doi: 10.1038/s12276-018-0126-x, PMID: 30115904 PMC6095905

[ref31] Chen S. Chen L. Qi Y. Xu J. Ge Q. Fan Y.. (2021). *Bifidobacterium adolescentis* regulates catalase activity and host metabolism and improves healthspan and lifespan in multiple species. Nat Aging 1, 991–1001. doi: 10.1038/s43587-021-00129-0, PMID: 37118342

[ref32] Chen P. Torralba M. Tan J. Embree M. Zengler K. Starkel P.. (2015). Supplementation of saturated long-chain fatty acids maintains intestinal eubiosis and reduces ethanol-induced liver injury in mice. Gastroenterology 148, 203–214.e16. doi: 10.1053/j.gastro.2014.09.014, PMID: 25239591 PMC4274236

[ref33] Chen X. Wu R. Li L. Zeng Y. Chen J. Wei M.. (2023). Pregnancy-induced changes to the gut microbiota drive macrophage pyroptosis and exacerbate septic inflammation. Immunity 56, 336–352.e9. doi: 10.1016/j.immuni.2023.01.015 36792573

[ref34] Chen L. Yang P. Hu L. Yang L. Chu H. Hou X. (2023). Modulating phenylalanine metabolism by *L. acidophilus* alleviates alcohol-related liver disease through enhancing intestinal barrier function. Cell Biosci. 13:24. doi: 10.1186/s13578-023-00974-z, PMID: 36739426 PMC9899391

[ref35] Chi L. Khan I. Lin Z. Zhang J. Lee M. Y. S. Leong W.. (2020). Fructo-oligosaccharides from Morinda officinalis remodeled gut microbiota and alleviated depression features in a stress rat model. Phytomedicine 67:153157. doi: 10.1016/j.phymed.2019.153157 31896054

[ref36] Chopyk D. M. Grakoui A. (2020). Contribution of the intestinal microbiome and gut barrier to hepatic disorders. Gastroenterology 159, 849–863. doi: 10.1053/j.gastro.2020.04.077, PMID: 32569766 PMC7502510

[ref37] Collins S. L. Stine J. G. Bisanz J. E. Okafor C. D. Patterson A. D. (2023). Bile acids and the gut microbiota: metabolic interactions and impacts on disease. Nat. Rev. Microbiol. 21, 236–247. doi: 10.1038/s41579-022-00805-x, PMID: 36253479 PMC12536349

[ref38] Costello S. P. Hughes P. A. Waters O. Bryant R. V. Vincent A. D. Blatchford P.. (2019). Effect of fecal microbiota transplantation on 8-week remission in patients with ulcerative colitis: a randomized clinical trial. JAMA 321, 156–164. doi: 10.1001/jama.2018.20046, PMID: 30644982 PMC6439766

[ref39] Couturier-Maillard A. Secher T. Rehman A. Normand S. De Arcangelis A. Haesler R.. (2013). NOD2-mediated dysbiosis predisposes mice to transmissible colitis and colorectal cancer. J. Clin. Invest. 123, 700–711. doi: 10.1172/JCI62236, PMID: 23281400 PMC3561825

[ref40] Cox T. O. Lundgren P. Nath K. Thaiss C. A. (2022). Metabolic control by the microbiome. Genome Med. 14:80. doi: 10.1186/s13073-022-01092-0, PMID: 35906678 PMC9338551

[ref41] Cruz B. Conceicao L. L. D. Mendes T. A. O. Ferreira C. Goncalves R. V. Peluzio M. (2020). Use of the synbiotic VSL#3 and yacon-based concentrate attenuates intestinal damage and reduces the abundance of Candidatus Saccharimonas in a colitis-associated carcinogenesis model. Food Res. Int. 137:109721. doi: 10.1016/j.foodres.2020.109721, PMID: 33233290

[ref42] Dalile B. Van Oudenhove L. Vervliet B. Verbeke K. (2019). The role of short-chain fatty acids in microbiota-gut-brain communication. Nat. Rev. Gastroenterol. Hepatol. 16, 461–478. doi: 10.1038/s41575-019-0157-3 31123355

[ref43] Darwish A. M. G. Allam M. G. Shokery E. S. Ayad E. H. E. (2022). Functional products fortified with probiotic LAB isolated from Egyptian dairy sources showed hypolipidemic effects in albino rats. PLoS One 17:e0263241. doi: 10.1371/journal.pone.0263241, PMID: 35235577 PMC8890634

[ref44] Das T. K. Ganesh B. P. (2023). Interlink between the gut microbiota and inflammation in the context of oxidative stress in Alzheimer's disease progression. Gut Microbes 15:2206504. doi: 10.1080/19490976.2023.2206504, PMID: 37127846 PMC10153019

[ref45] Datta S. Cano M. Ebrahimi K. Wang L. Handa J. T. (2017). The impact of oxidative stress and inflammation on RPE degeneration in non-neovascular AMD. Prog. Retin. Eye Res. 60, 201–218. doi: 10.1016/j.preteyeres.2017.03.002, PMID: 28336424 PMC5600827

[ref46] De Salvo C. Buela K. A. Creyns B. Corridoni D. Rana N. Wargo H. L.. (2021). NOD2 drives early IL-33-dependent expansion of group 2 innate lymphoid cells during Crohn's disease-like ileitis. J. Clin. Invest. 131:e140624. doi: 10.1172/JCI140624, PMID: 33444291 PMC7919719

[ref47] de Vos W. M. Tilg H. Van Hul M. Cani P. D. (2022). Gut microbiome and health: mechanistic insights. Gut 71, 1020–1032. doi: 10.1136/gutjnl-2021-326789, PMID: 35105664 PMC8995832

[ref48] Denson L. A. Jurickova I. Karns R. Shaw K. A. Cutler D. J. Okou D. T.. (2018). Clinical and genomic correlates of neutrophil reactive oxygen species production in pediatric patients with Crohn's disease. Gastroenterology 154, 2097–2110. doi: 10.1053/j.gastro.2018.02.016, PMID: 29454792 PMC5985211

[ref49] Dey P. Ray Chaudhuri S. (2023). The opportunistic nature of gut commensal microbiota. Crit. Rev. Microbiol. 49, 739–763. doi: 10.1080/1040841X.2022.2133987 36256871

[ref50] Dsouza M. Menon R. Crossette E. Bhattarai S. K. Schneider J. Kim Y. G.. (2022). Colonization of the live biotherapeutic product VE303 and modulation of the microbiota and metabolites in healthy volunteers. Cell Host Microbe 30, 583–598.e8. doi: 10.1016/j.chom.2022.03.016, PMID: 35421353

[ref51] Enright E. F. Griffin B. T. Gahan C. G. M. Joyce S. A. (2018). Microbiome-mediated bile acid modification: role in intestinal drug absorption and metabolism. Pharmacol. Res. 133, 170–186. doi: 10.1016/j.phrs.2018.04.009, PMID: 29660405

[ref52] Erttmann S. F. Swacha P. Aung K. M. Brindefalk B. Jiang H. Hartlova A.. (2022). The gut microbiota prime systemic antiviral immunity via the cGAS-STING-IFN-I axis. Immunity 55, 847–861.e10. doi: 10.1016/j.immuni.2022.04.006, PMID: 35545033

[ref53] Evans C. R. Kempes C. P. Price-Whelan A. Dietrich L. E. P. (2020). Metabolic heterogeneity and cross-feeding in bacterial multicellular systems. Trends Microbiol. 28, 732–743. doi: 10.1016/j.tim.2020.03.008, PMID: 32781027 PMC7419710

[ref54] Fan Y. Pedersen O. (2021). Gut microbiota in human metabolic health and disease. Nat. Rev. Microbiol. 19, 55–71. doi: 10.1038/s41579-020-0433-9 32887946

[ref55] Fang D. Xu T. Sun J. Shi J. Li F. Yin Y.. (2023). Nicotinamide mononucleotide ameliorates sleep deprivation-induced gut microbiota Dysbiosis and restores colonization resistance against intestinal infections. Adv Sci (Weinh) 10:e2207170. doi: 10.1002/advs.202207170, PMID: 36698264 PMC10037695

[ref56] Fassarella M. Blaak E. E. Penders J. Nauta A. Smidt H. Zoetendal E. G. (2021). Gut microbiome stability and resilience: elucidating the response to perturbations in order to modulate gut health. Gut 70, 595–605. doi: 10.1136/gutjnl-2020-321747, PMID: 33051190

[ref57] Ferrand A. Al Nabhani Z. Tapias N. S. Mas E. Hugot J. P. Barreau F. (2019). NOD2 expression in intestinal epithelial cells protects toward the development of inflammation and associated carcinogenesis. Cell Mol. Gastroenterol. Hepatol. 7, 357–369. doi: 10.1016/j.jcmgh.2018.10.009 30704984 PMC6357788

[ref58] Ford A. C. Harris L. A. Lacy B. E. Quigley E. M. M. Moayyedi P. (2018). Systematic review with meta-analysis: the efficacy of prebiotics, probiotics, synbiotics and antibiotics in irritable bowel syndrome. Aliment. Pharmacol. Ther. 48, 1044–1060. doi: 10.1111/apt.15001 30294792

[ref59] Fung T. C. Vuong H. E. Luna C. D. G. Pronovost G. N. Aleksandrova A. A. Riley N. G.. (2019). Intestinal serotonin and fluoxetine exposure modulate bacterial colonization in the gut. Nat. Microbiol. 4, 2064–2073. doi: 10.1038/s41564-019-0540-4, PMID: 31477894 PMC6879823

[ref60] Gao J. Wang L. Jiang J. Xu Q. Zeng N. Lu B.. (2023). A probiotic bi-functional peptidoglycan hydrolase sheds NOD2 ligands to regulate gut homeostasis in female mice. Nat. Commun. 14:3338. doi: 10.1038/s41467-023-38950-3, PMID: 37286542 PMC10247697

[ref61] Garo L. P. Ajay A. K. Fujiwara M. Gabriely G. Raheja R. Kuhn C.. (2021). MicroRNA-146a limits tumorigenic inflammation in colorectal cancer. Nat. Commun. 12:2419. doi: 10.1038/s41467-021-22641-y, PMID: 33893298 PMC8065171

[ref62] Ghosh T. S. Rampelli S. Jeffery I. B. Santoro A. Neto M. Capri M.. (2020). Mediterranean diet intervention alters the gut microbiome in older people reducing frailty and improving health status: the NU-AGE 1-year dietary intervention across five European countries. Gut 69, 1218–1228. doi: 10.1136/gutjnl-2019-319654, PMID: 32066625 PMC7306987

[ref63] Goes V. Monte D. F. M. Saraiva M. M. S. Maria de Almeida A. Cabrera J. M. Rodrigues Alves L. B.. (2022). Salmonella Heidelberg side-step gene loss of respiratory requirements in chicken infection model. Microb. Pathog. 171:105725. doi: 10.1016/j.micpath.2022.105725, PMID: 36007847

[ref64] Goguyer-Deschaumes R. Waeckel L. Killian M. Rochereau N. Paul S. (2022). Metabolites and secretory immunoglobulins: messengers and effectors of the host-microbiota intestinal equilibrium. Trends Immunol. 43, 63–77. doi: 10.1016/j.it.2021.11.005, PMID: 34848167

[ref65] Goldenberg J. Z. Yap C. Lytvyn L. Lo C. K. Beardsley J. Mertz D.. (2017). Probiotics for the prevention of *Clostridium difficile*-associated diarrhea in adults and children. Cochrane Database Syst. Rev. 12:CD006095. doi: 10.1002/14651858.CD006095.pub4, PMID: 29257353 PMC6486212

[ref66] Gopalakrishnan V. Helmink B. A. Spencer C. N. Reuben A. Wargo J. A. (2018). The influence of the gut microbiome on Cancer, immunity, and Cancer immunotherapy. Cancer Cell 33, 570–580. doi: 10.1016/j.ccell.2018.03.015, PMID: 29634945 PMC6529202

[ref67] Gracie D. J. Hamlin P. J. Ford A. C. (2019). The influence of the brain-gut axis in inflammatory bowel disease and possible implications for treatment. Lancet Gastroenterol. Hepatol. 4, 632–642. doi: 10.1016/S2468-1253(19)30089-5, PMID: 31122802

[ref68] Grosheva I. Zheng D. Levy M. Polansky O. Lichtenstein A. Golani O.. (2020). High-throughput screen identifies host and microbiota regulators of intestinal barrier function. Gastroenterology 159, 1807–1823. doi: 10.1053/j.gastro.2020.07.003, PMID: 32653496

[ref69] Guery B. Galperine T. Barbut F. (2019). *Clostridioides difficile*: diagnosis and treatments. BMJ 366:l4609. doi: 10.1136/bmj.l4609 31431428

[ref70] Guo X. Dong Z. Li Q. Wan D. Zhong J. Dongzhi D.. (2022). Flavonoids from Rhododendron nivale hook. F delay aging via modulation of gut microbiota and glutathione metabolism. Phytomedicine 104:154270. doi: 10.1016/j.phymed.2022.154270, PMID: 35760023

[ref71] Gupta A. Khanna S. (2017). Fecal microbiota transplantation. JAMA 318:102. doi: 10.1001/jama.2017.6466 28672320

[ref72] Haran J. P. McCormick B. A. (2021). Aging, frailty, and the microbiome-how Dysbiosis influences human aging and disease. Gastroenterology 160, 507–523. doi: 10.1053/j.gastro.2020.09.060, PMID: 33307030 PMC7856216

[ref73] Hayase E. Jenq R. R. (2021). Role of the intestinal microbiome and microbial-derived metabolites in immune checkpoint blockade immunotherapy of cancer. Genome Med. 13:107. doi: 10.1186/s13073-021-00923-w, PMID: 34162429 PMC8220726

[ref74] Hill C. Guarner F. Reid G. Gibson G. R. Merenstein D. J. Pot B.. (2014). Expert consensus document the international scientific Association for Probiotics and Prebiotics consensus statement on the scope and appropriate use of the term probiotic. Nat. Rev. Gastroenterol. Hepatol. 11, 506–514. doi: 10.1038/nrgastro.2014.66, PMID: 24912386

[ref75] Hodgkinson K. El Abbar F. Dobranowski P. Manoogian J. Butcher J. Figeys D.. (2023). Butyrate's role in human health and the current progress towards its clinical application to treat gastrointestinal disease. Clin. Nutr. 42, 61–75. doi: 10.1016/j.clnu.2022.10.024, PMID: 36502573

[ref76] Honda M. Kubes P. (2018). Neutrophils and neutrophil extracellular traps in the liver and gastrointestinal system. Nat. Rev. Gastroenterol. Hepatol. 15, 206–221. doi: 10.1038/nrgastro.2017.183 29382950

[ref77] Hopfner K. P. Hornung V. (2020). Molecular mechanisms and cellular functions of cGAS-STING signalling. Nat. Rev. Mol. Cell Biol. 21, 501–521. doi: 10.1038/s41580-020-0244-x 32424334

[ref78] Hou Y. Li X. Liu X. Zhang Y. Zhang W. Man C.. (2019). Transcriptomic responses of Caco-2 cells to *Lactobacillus rhamnosus* GG and *Lactobacillus plantarum* J26 against oxidative stress. J. Dairy Sci. 102, 7684–7696. doi: 10.3168/jds.2019-16332, PMID: 31255276

[ref79] Hu B. Jin C. Li H. B. Tong J. Ouyang X. Cetinbas N. M.. (2016). The DNA-sensing AIM2 inflammasome controls radiation-induced cell death and tissue injury. Science 354, 765–768. doi: 10.1126/science.aaf7532, PMID: 27846608 PMC5640175

[ref80] Huycke M. M. Moore D. R. (2002). In vivo production of hydroxyl radical by *Enterococcus faecalis* colonizing the intestinal tract using aromatic hydroxylation. Free Radic. Biol. Med. 33, 818–826. doi: 10.1016/S0891-5849(02)00977-2, PMID: 12208369

[ref81] Imhann F. Vich Vila A. Bonder M. J. Fu J. Gevers D. Visschedijk M. C.. (2018). Interplay of host genetics and gut microbiota underlying the onset and clinical presentation of inflammatory bowel disease. Gut 67, 108–119. doi: 10.1136/gutjnl-2016-312135, PMID: 27802154 PMC5699972

[ref82] Intarajak T. Udomchaiprasertkul W. Bunyoo C. Yimnoon J. Soonklang K. Wiriyaukaradecha K.. (2019). Genetic aberration analysis in Thai colorectal adenoma and early-stage adenocarcinoma patients by whole-exome sequencing. Cancers (Basel) 11:977. doi: 10.3390/cancers11070977, PMID: 31336886 PMC6679221

[ref83] Jabot C. Daniele G. Giroud B. Tchamitchian S. Belzunces L. P. Casabianca H.. (2016). Detection and quantification of boscalid and its metabolites in honeybees. Chemosphere 156, 245–251. doi: 10.1016/j.chemosphere.2016.04.135, PMID: 27179242

[ref84] Jackson D. N. Theiss A. L. (2020). Gut bacteria signaling to mitochondria in intestinal inflammation and cancer. Gut Microbes 11, 285–304. doi: 10.1080/19490976.2019.1592421, PMID: 30913966 PMC7524274

[ref85] Jakubczyk K. Druzga A. Katarzyna J. Skonieczna-Zydecka K. (2020). Antioxidant potential of curcumin – a meta-analysis of randomized clinical trials. Antioxidants (Basel) 9:1092. doi: 10.3390/antiox9111092 33172016 PMC7694612

[ref86] Janney A. Powrie F. Mann E. H. (2020). Host-microbiota maladaptation in colorectal cancer. Nature 585, 509–517. doi: 10.1038/s41586-020-2729-3, PMID: 32968260

[ref87] Jones R. M. Neish A. S. (2021). Gut microbiota in intestinal and liver disease. Annu. Rev. Pathol. 16, 251–275. doi: 10.1146/annurev-pathol-030320-095722 33234022

[ref88] Kamada N. Chen G. Y. Inohara N. Nunez G. (2013). Control of pathogens and pathobionts by the gut microbiota. Nat. Immunol. 14, 685–690. doi: 10.1038/ni.2608, PMID: 23778796 PMC4083503

[ref89] Kapil V. Khambata R. S. Jones D. A. Rathod K. Primus C. Massimo G.. (2020). The noncanonical pathway for in vivo nitric oxide generation: the nitrate-nitrite-nitric oxide pathway. Pharmacol. Rev. 72, 692–766. doi: 10.1124/pr.120.019240, PMID: 32576603

[ref90] Kastl A. J. Jr. Terry N. A. Wu G. D. Albenberg L. G. (2020). The structure and function of the human small intestinal microbiota: current understanding and future directions. Cell. Mol. Gastroenterol. Hepatol. 9, 33–45. doi: 10.1016/j.jcmgh.2019.07.006, PMID: 31344510 PMC6881639

[ref91] Khan S. Imran A. Malik A. Chaudhary A. A. Rub A. Jan A. T.. (2019). Bacterial imbalance and gut pathologies: association and contribution of *E. coli* in inflammatory bowel disease. Crit. Rev. Clin. Lab. Sci. 56, 1–17. doi: 10.1080/10408363.2018.1517144, PMID: 30373492

[ref92] Kim C. H. (2021). Control of lymphocyte functions by gut microbiota-derived short-chain fatty acids. Cell. Mol. Immunol. 18, 1161–1171. doi: 10.1038/s41423-020-00625-0, PMID: 33850311 PMC8093302

[ref93] Kirk J. A. Gebhart D. Buckley A. M. Lok S. Scholl D. Douce G. R.. (2017). New class of precision antimicrobials redefines role of *Clostridium difficile* S-layer in virulence and viability. Sci. Transl. Med. 9:eaah6813. doi: 10.1126/scitranslmed.aah6813, PMID: 28878013 PMC5603275

[ref94] Kitamoto S. Nagao-Kitamoto H. Jiao Y. Gillilland M. G. 3rd Hayashi A. Imai J.. (2020). The intermucosal connection between the mouth and gut in commensal Pathobiont-driven colitis. Cell 182, 447–462.e14. doi: 10.1016/j.cell.2020.05.048, PMID: 32758418 PMC7414097

[ref95] Koren E. Fuchs Y. (2021). Modes of regulated cell death in Cancer. Cancer Discov. 11, 245–265. doi: 10.1158/2159-8290.CD-20-0789 33462123

[ref96] Langdon A. Schwartz D. J. Bulow C. Sun X. Hink T. Reske K. A.. (2021). Microbiota restoration reduces antibiotic-resistant bacteria gut colonization in patients with recurrent *Clostridioides difficile* infection from the open-label PUNCH CD study. Genome Med. 13:28. doi: 10.1186/s13073-021-00843-9, PMID: 33593430 PMC7888090

[ref97] Lavelle A. Sokol H. (2020). Gut microbiota-derived metabolites as key actors in inflammatory bowel disease. Nat. Rev. Gastroenterol. Hepatol. 17, 223–237. doi: 10.1038/s41575-019-0258-z, PMID: 32076145

[ref98] Lecuyer E. Rakotobe S. Lengline-Garnier H. Lebreton C. Picard M. Juste C.. (2014). Segmented filamentous bacterium uses secondary and tertiary lymphoid tissues to induce gut IgA and specific T helper 17 cell responses. Immunity 40, 608–620. doi: 10.1016/j.immuni.2014.03.009, PMID: 24745335

[ref99] Leta V. Ray Chaudhuri K. Milner O. Chung-Faye G. Metta V. Pariante C. M.. (2021). Neurogenic and anti-inflammatory effects of probiotics in Parkinson's disease: a systematic review of preclinical and clinical evidence. Brain Behav. Immun. 98, 59–73. doi: 10.1016/j.bbi.2021.07.026, PMID: 34364965

[ref100] Li Q. Cui Y. Xu B. Wang Y. Lv F. Li Z.. (2021). Main active components of Jiawei Gegen Qinlian decoction protects against ulcerative colitis under different dietary environments in a gut microbiota-dependent manner. Pharmacol. Res. 170:105694. doi: 10.1016/j.phrs.2021.105694, PMID: 34087350

[ref101] Li B. Du P. Du Y. Zhao D. Cai Y. Yang Q.. (2021). Luteolin alleviates inflammation and modulates gut microbiota in ulcerative colitis rats. Life Sci. 269:119008. doi: 10.1016/j.lfs.2020.119008, PMID: 33434535

[ref102] Li L. Jin P. Guan Y. Luo M. Wang Y. He B.. (2022). Exploiting polyphenol-mediated redox reorientation in cancer therapy. Pharmaceuticals (Basel) 15:1540. doi: 10.3390/ph15121540, PMID: 36558995 PMC9787032

[ref103] Li J. Li Q. Gao N. Wang Z. Li F. Li J.. (2021). Exopolysaccharides produced by *Lactobacillus rhamnosus* GG alleviate hydrogen peroxide-induced intestinal oxidative damage and apoptosis through the Keap1/Nrf2 and Bax/Bcl-2 pathways in vitro. Food Funct. 12, 9632–9641. doi: 10.1039/D1FO00277E, PMID: 34664577

[ref104] Li L. Peng P. Ding N. Jia W. Huang C. Tang Y. (2023). Oxidative stress, inflammation, gut Dysbiosis: what can polyphenols do in inflammatory bowel disease? Antioxidants (Basel) 12:967. doi: 10.3390/antiox12040967 37107341 PMC10135842

[ref105] Li T. Zhang T. Gao H. Liu R. Gu M. Yang Y.. (2021). Tempol ameliorates polycystic ovary syndrome through attenuating intestinal oxidative stress and modulating of gut microbiota composition-serum metabolites interaction. Redox Biol. 41:101886. doi: 10.1016/j.redox.2021.101886, PMID: 33592539 PMC7896192

[ref106] Limketkai B. N. Akobeng A. K. Gordon M. Adepoju A. A. (2020). Probiotics for induction of remission in Crohn's disease. Cochrane Database Syst. Rev. 7:CD006634. doi: 10.1002/14651858.CD006634.pub3, PMID: 32678465 PMC7389339

[ref107] Lin Z. Chen L. Cheng M. Zhu F. Yang X. Zhao W.. (2022). Cortex periplocae modulates the gut microbiota to restrict colitis and colitis-associated colorectal cancer via suppression of pathogenic Th17 cells. Biomed. Pharmacother. 153:113399. doi: 10.1016/j.biopha.2022.113399 35834986

[ref108] Lipska K. Gumieniczek A. Filip A. A. (2020). Anticonvulsant valproic acid and other short-chain fatty acids as novel anticancer therapeutics: possibilities and challenges. Acta Pharma. 70, 291–301. doi: 10.2478/acph-2020-0021, PMID: 32074065

[ref109] Litichevskiy L. Thaiss C. A. (2022). The oscillating gut microbiome and its effects on host circadian biology. Annu. Rev. Nutr. 42, 145–164. doi: 10.1146/annurev-nutr-062320-111321, PMID: 35576592

[ref110] Litvak Y. Byndloss M. X. Tsolis R. M. Baumler A. J. (2017). Dysbiotic Proteobacteria expansion: a microbial signature of epithelial dysfunction. Curr. Opin. Microbiol. 39, 1–6. doi: 10.1016/j.mib.2017.07.003, PMID: 28783509

[ref111] Liu J. R. Miao H. Deng D. Q. Vaziri N. D. Li P. Zhao Y. Y. (2021). Gut microbiota-derived tryptophan metabolism mediates renal fibrosis by aryl hydrocarbon receptor signaling activation. Cell. Mol. Life Sci. 78, 909–922. doi: 10.1007/s00018-020-03645-1, PMID: 32965514 PMC11073292

[ref112] Lomasney K. W. Cryan J. F. Hyland N. P. (2014). Converging effects of a Bifidobacterium and Lactobacillus probiotic strain on mouse intestinal physiology. Am. J. Physiol. Gastrointest. Liver Physiol. 307, G241–G247. doi: 10.1152/ajpgi.00401.2013, PMID: 24852567

[ref113] Luca M. Di Mauro M. Di Mauro M. Luca A. (2019). Gut microbiota in Alzheimer's disease, depression, and type 2 diabetes mellitus: the role of oxidative stress. Oxidative Med. Cell. Longev. 2019:4730539. doi: 10.1155/2019/4730539 PMC6501164 31178961

[ref114] Luceri C. Bigagli E. Agostiniani S. Giudici F. Zambonin D. Scaringi S.. (2019). Analysis of oxidative stress-related markers in Crohn's disease patients at surgery and correlations with clinical findings. Antioxidants (Basel) 8:378. doi: 10.3390/antiox8090378 31489956 PMC6771139

[ref115] Lundberg J. O. Weitzberg E. Gladwin M. T. (2008). The nitrate-nitrite-nitric oxide pathway in physiology and therapeutics. Nat. Rev. Drug Discov. 7, 156–167. doi: 10.1038/nrd2466 18167491

[ref116] Lv B. Huang X. Lijia C. Ma Y. Bian M. Li Z.. (2023). Heat shock potentiates aminoglycosides against gram-negative bacteria by enhancing antibiotic uptake, protein aggregation, and ROS. Proc. Natl. Acad. Sci. USA 120:e2217254120. doi: 10.1073/pnas.2217254120, PMID: 36917671 PMC10041086

[ref117] Lyu M. Suzuki H. Kang L. Gaspal F. Zhou W. Goc J.. (2022). ILC3s select microbiota-specific regulatory T cells to establish tolerance in the gut. Nature 610, 744–751. doi: 10.1038/s41586-022-05141-x, PMID: 36071169 PMC9613541

[ref118] Ma K. Bai Y. Li J. Ren Z. Li J. Zhang J.. (2022). *Lactobacillus rhamnosus* GG ameliorates deoxynivalenol-induced kidney oxidative damage and mitochondrial injury in weaned piglets. Food Funct. 13, 3905–3916. doi: 10.1039/D2FO00185C 35285834

[ref119] Ma P. Mo R. Liao H. Qiu C. Wu G. Yang C.. (2022). Gut microbiota depletion by antibiotics ameliorates somatic neuropathic pain induced by nerve injury, chemotherapy, and diabetes in mice. J. Neuroinflammation 19:169. doi: 10.1186/s12974-022-02523-w, PMID: 35764988 PMC9237999

[ref120] Macia L. Tan J. Vieira A. T. Leach K. Stanley D. Luong S.. (2015). Metabolite-sensing receptors GPR43 and GPR109A facilitate dietary fibre-induced gut homeostasis through regulation of the inflammasome. Nat. Commun. 6:6734. doi: 10.1038/ncomms7734, PMID: 25828455

[ref121] Maier L. Goemans C. V. Wirbel J. Kuhn M. Eberl C. Pruteanu M.. (2021). Unravelling the collateral damage of antibiotics on gut bacteria. Nature 599, 120–124. doi: 10.1038/s41586-021-03986-2, PMID: 34646011 PMC7612847

[ref122] Marotto D. Atzeni F. Ardizzone S. Monteleone G. Giorgi V. Sarzi-Puttini P. (2020). Extra-intestinal manifestations of inflammatory bowel diseases. Pharmacol. Res. 161:105206. doi: 10.1016/j.phrs.2020.105206 32998068

[ref123] Martens E. C. Neumann M. Desai M. S. (2018). Interactions of commensal and pathogenic microorganisms with the intestinal mucosal barrier. Nat. Rev. Microbiol. 16, 457–470. doi: 10.1038/s41579-018-0036-x, PMID: 29904082

[ref124] Martins F. H. Rajan A. Carter H. E. Baniasadi H. R. Maresso A. W. Sperandio V. (2022). Interactions between Enterohemorrhagic *Escherichia coli* (EHEC) and gut commensals at the Interface of human colonoids. mBio 13:e0132122. doi: 10.1128/mbio.01321-22 35638758 PMC9239246

[ref125] Mazenc A. Mervant L. Maslo C. Lencina C. Bezirard V. Leveque M.. (2022). Maternal heme-enriched diet promotes a gut pro-oxidative status associated with microbiota alteration, gut leakiness and glucose intolerance in mice offspring. Redox Biol. 53:102333. doi: 10.1016/j.redox.2022.102333, PMID: 35588638 PMC9119830

[ref126] McCulloch J. A. Davar D. Rodrigues R. R. Badger J. H. Fang J. R. Cole A. M.. (2022). Intestinal microbiota signatures of clinical response and immune-related adverse events in melanoma patients treated with anti-PD-1. Nat. Med. 28, 545–556. doi: 10.1038/s41591-022-01698-2, PMID: 35228752 PMC10246505

[ref127] McDonnell L. Gilkes A. Ashworth M. Rowland V. Harries T. H. Armstrong D.. (2021). Association between antibiotics and gut microbiome dysbiosis in children: systematic review and meta-analysis. Gut Microbes 13, 1–18. doi: 10.1080/19490976.2020.1870402, PMID: 33651651 PMC7928022

[ref128] Mei F. Wu M. Zhao L. Hu K. Gao Q. Chen F.. (2022). Probiotics for the prevention of Hirschsprung-associated enterocolitis. Cochrane Database Syst. Rev. 4:CD013714. doi: 10.1002/14651858.CD013714.pub2, PMID: 35470864 PMC9039968

[ref129] Metta V. Leta V. Mrudula K. R. Prashanth L. K. Goyal V. Borgohain R.. (2022). Gastrointestinal dysfunction in Parkinson's disease: molecular pathology and implications of gut microbiome, probiotics, and fecal microbiota transplantation. J. Neurol. 269, 1154–1163. doi: 10.1007/s00415-021-10567-w, PMID: 33881598

[ref130] Michaudel C. Sokol H. (2020). The gut microbiota at the Service of Immunometabolism. Cell Metab. 32, 514–523. doi: 10.1016/j.cmet.2020.09.004, PMID: 32946809

[ref131] Mirji G. Worth A. Bhat S. A. El Sayed M. Kannan T. Goldman A. R.. (2022). The microbiome-derived metabolite TMAO drives immune activation and boosts responses to immune checkpoint blockade in pancreatic cancer. Sci Immunol 7:eabn0704. doi: 10.1126/sciimmunol.abn0704, PMID: 36083892 PMC9925043

[ref132] Mitra S. Dash R. Nishan A. A. Habiba S. U. Moon I. S. (2022). Brain modulation by the gut microbiota: from disease to therapy. J. Adv. Res. 53, 153–173. doi: 10.1016/j.jare.2022.12.001 36496175 PMC10658262

[ref133] Mitrea L. Medeleanu M. Pop C. R. Rotar A. M. Vodnar D. C. (2023). Biotics (pre-, pro-, Post-) and uremic toxicity: implications, mechanisms, and possible therapies. Toxins (Basel) 15:548. doi: 10.3390/toxins15090548, PMID: 37755974 PMC10535688

[ref134] Mitrea L. Nemes S. A. Szabo K. Teleky B. E. Vodnar D. C. (2022). Guts imbalance imbalances the brain: a review of gut microbiota association with neurological and psychiatric disorders. Front Med (Lausanne) 9:813204. doi: 10.3389/fmed.2022.813204, PMID: 35433746 PMC9009523

[ref135] Miyauchi E. Kim S. W. Suda W. Kawasumi M. Onawa S. Taguchi-Atarashi N.. (2020). Gut microorganisms act together to exacerbate inflammation in spinal cords. Nature 585, 102–106. doi: 10.1038/s41586-020-2634-9, PMID: 32848245

[ref136] Moens F. Duysburgh C. van den Abbeele P. Morera M. Marzorati M. (2019). *Lactobacillus rhamnosus* GG and *Saccharomyces cerevisiae* boulardii exert synergistic antipathogenic activity in vitro against enterotoxigenic *Escherichia coli*. Benef Microbes 10, 923–935. doi: 10.3920/BM2019.0064, PMID: 31965838

[ref137] Monteagudo-Mera A. Arthur J. C. Jobin C. Keku T. Bruno-Barcena J. M. Azcarate-Peril M. A. (2016). High purity galacto-oligosaccharides enhance specific Bifidobacterium species and their metabolic activity in the mouse gut microbiome. Benef Microbes 7, 247–264. doi: 10.3920/BM2015.0114, PMID: 26839072 PMC4974821

[ref138] Morais L. H. Schreiber H. L. T. Mazmanian S. K. (2021). The gut microbiota-brain axis in behaviour and brain disorders. Nat. Rev. Microbiol. 19, 241–255. doi: 10.1038/s41579-020-00460-0 33093662

[ref139] Morana O. Wood W. Gregory C. D. (2022). The apoptosis paradox in cancer. Int. J. Mol. Sci. 23:1328. doi: 10.3390/ijms23031328, PMID: 35163253 PMC8836235

[ref140] Mossad O. Batut B. Yilmaz B. Dokalis N. Mezo C. Nent E.. (2022). Gut microbiota drives age-related oxidative stress and mitochondrial damage in microglia via the metabolite N(6)-carboxymethyllysine. Nat. Neurosci. 25, 295–305. doi: 10.1038/s41593-022-01027-3, PMID: 35241804

[ref141] Muendlein H. I. Connolly W. M. Magri Z. Jetton D. Smirnova I. Degterev A.. (2022). ZBP1 promotes inflammatory responses downstream of TLR3/TLR4 via timely delivery of RIPK1 to TRIF. Proc. Natl. Acad. Sci. USA 119:e2113872119. doi: 10.1073/pnas.2113872119, PMID: 35666872 PMC9214535

[ref142] Nabavi-Rad A. Sadeghi A. Asadzadeh Aghdaei H. Yadegar A. Smith S. M. Zali M. R. (2022). The double-edged sword of probiotic supplementation on gut microbiota structure in *Helicobacter pylori* management. Gut Microbes 14:2108655. doi: 10.1080/19490976.2022.2108655, PMID: 35951774 PMC9373750

[ref143] Nakkarach A. Foo H. L. Song A. A. Mutalib N. E. A. Nitisinprasert S. Withayagiat U. (2021). Anti-cancer and anti-inflammatory effects elicited by short chain fatty acids produced by *Escherichia coli* isolated from healthy human gut microbiota. Microb. Cell Factories 20:36. doi: 10.1186/s12934-020-01477-z, PMID: 33546705 PMC7863513

[ref144] Nicolucci A. C. Hume M. P. Martinez I. Mayengbam S. Walter J. Reimer R. A. (2017). Prebiotics reduce body fat and Alter intestinal microbiota in children who are overweight or with obesity. Gastroenterology 153, 711–722. doi: 10.1053/j.gastro.2017.05.055 28596023

[ref145] Nogal A. Valdes A. M. Menni C. (2021). The role of short-chain fatty acids in the interplay between gut microbiota and diet in cardio-metabolic health. Gut Microbes 13, 1–24. doi: 10.1080/19490976.2021.1897212, PMID: 33764858 PMC8007165

[ref146] Ooijevaar R. E. Terveer E. M. Verspaget H. W. Kuijper E. J. Keller J. J. (2019). Clinical application and potential of fecal microbiota transplantation. Annu. Rev. Med. 70, 335–351. doi: 10.1146/annurev-med-111717-122956 30403550

[ref147] Ortega M. A. Alvarez-Mon M. A. Garcia-Montero C. Fraile-Martinez O. Monserrat J. Martinez-Rozas L.. (2023). Microbiota-gut-brain axis mechanisms in the complex network of bipolar disorders: potential clinical implications and translational opportunities. Mol. Psychiatry 28, 2645–2673. doi: 10.1038/s41380-023-01964-w, PMID: 36707651 PMC10615769

[ref148] Osbelt L. Wende M. Almasi E. Derksen E. Muthukumarasamy U. Lesker T. R.. (2021). *Klebsiella oxytoca* causes colonization resistance against multidrug-resistant *K. pneumoniae* in the gut via cooperative carbohydrate competition. Cell Host Microbe 29, 1663–1679.e7. doi: 10.1016/j.chom.2021.09.003, PMID: 34610293

[ref149] Pan S. Guo Y. Hong F. Xu P. Zhai Y. (2022). Therapeutic potential of melatonin in colorectal cancer: focus on lipid metabolism and gut microbiota. Biochim. Biophys. Acta Mol. basis Dis. 1868:166281. doi: 10.1016/j.bbadis.2021.166281, PMID: 34610472

[ref150] Park E. S. Jeon H. Lee N. Yu J. Park H. W. Satoh T.. (2023). TDAG51 promotes transcription factor FoxO1 activity during LPS-induced inflammatory responses. EMBO J. 42:e111867. doi: 10.15252/embj.2022111867, PMID: 37203866 PMC10308371

[ref151] Parker A. Fonseca S. Carding S. R. (2020). Gut microbes and metabolites as modulators of blood-brain barrier integrity and brain health. Gut Microbes 11, 135–157. doi: 10.1080/19490976.2019.1638722, PMID: 31368397 PMC7053956

[ref152] Parker A. Romano S. Ansorge R. Aboelnour A. Le Gall G. Savva G. M.. (2022). Fecal microbiota transfer between young and aged mice reverses hallmarks of the aging gut, eye, and brain. Microbiome 10:68. doi: 10.1186/s40168-022-01243-w, PMID: 35501923 PMC9063061

[ref153] Pellon A. Barriales D. Pena-Cearra A. Castelo-Careaga J. Palacios A. Lopez N.. (2021). The commensal bacterium Lactiplantibacillus plantarum imprints innate memory-like responses in mononuclear phagocytes. Gut Microbes 13:1939598. doi: 10.1080/19490976.2021.1939598, PMID: 34224309 PMC8259724

[ref154] Pleguezuelos-Manzano C. Puschhof J. Huber A. R. Van Hoeck A. Wood H. M. Nomburg J.. (2020). Mutational signature in colorectal cancer caused by genotoxic pks(+) *E. coli*. Nature 580, 269–273. doi: 10.1038/s41586-020-2080-8 32106218 PMC8142898

[ref155] Pujo J. Petitfils C. Le Faouder P. Eeckhaut V. Payros G. Maurel S.. (2021). Bacteria-derived long chain fatty acid exhibits anti-inflammatory properties in colitis. Gut 70, 1088–1097. doi: 10.1136/gutjnl-2020-321173, PMID: 32978245

[ref156] Puschhof J. Sears C. L. (2022). Microbial metabolites damage DNA. Science 378, 358–359. doi: 10.1126/science.ade6952 36302018

[ref157] Querdasi F. R. Enders C. Karnani N. Broekman B. Yap Seng C. Gluckman P. D.. (2023). Multigenerational adversity impacts on human gut microbiome composition and socioemotional functioning in early childhood. Proc. Natl. Acad. Sci. USA 120:e2213768120. doi: 10.1073/pnas.2213768120, PMID: 37463211 PMC10372691

[ref158] Quigley E. M. M. (2019). Prebiotics and probiotics in digestive health. Clin. Gastroenterol. Hepatol. 17, 333–344. doi: 10.1016/j.cgh.2018.09.028 30267869

[ref159] Ran M. Li Q. Xin Y. Ma S. Zhao R. Wang M.. (2022). Rhodaneses minimize the accumulation of cellular sulfane sulfur to avoid disulfide stress during sulfide oxidation in bacteria. Redox Biol. 53:102345. doi: 10.1016/j.redox.2022.102345, PMID: 35653932 PMC9163753

[ref160] Ray S. Abugable A. A. Parker J. Liversidge K. Palminha N. M. Liao C.. (2022). A mechanism for oxidative damage repair at gene regulatory elements. Nature 609, 1038–1047. doi: 10.1038/s41586-022-05217-8, PMID: 36171374

[ref161] Rivera-Chavez F. Zhang L. F. Faber F. Lopez C. A. Byndloss M. X. Olsan E. E.. (2016). Depletion of butyrate-producing Clostridia from the gut microbiota drives an aerobic luminal expansion of Salmonella. Cell Host Microbe 19, 443–454. doi: 10.1016/j.chom.2016.03.004, PMID: 27078066 PMC4832419

[ref162] Rogers A. P. Mileto S. J. Lyras D. (2023). Impact of enteric bacterial infections at and beyond the epithelial barrier. Nat. Rev. Microbiol. 21, 260–274. doi: 10.1038/s41579-022-00794-x, PMID: 36175770

[ref163] Rogers A. W. L. Tsolis R. M. Baumler A. J. (2021). Salmonella versus the microbiome. Microbiol. Mol. Biol. Rev. 85:e00027-19. doi: 10.1128/MMBR.00027-19, PMID: 33361269 PMC8549850

[ref164] Sanders M. E. Merenstein D. J. Reid G. Gibson G. R. Rastall R. A. (2019). Probiotics and prebiotics in intestinal health and disease: from biology to the clinic. Nat. Rev. Gastroenterol. Hepatol. 16, 605–616. doi: 10.1038/s41575-019-0173-3 31296969

[ref165] Sarkar A. Harty S. Moeller A. H. Klein S. L. Erdman S. E. Friston K. J.. (2021). The gut microbiome as a biomarker of differential susceptibility to SARS-CoV-2. Trends Mol. Med. 27, 1115–1134. doi: 10.1016/j.molmed.2021.09.009, PMID: 34756546 PMC8492747

[ref166] Sassone-Corsi M. Azriel S. Simon A. Ramanan D. Ortiz-Lopez A. Chen F.. (2022). Sequestration of gut pathobionts in intraluminal casts, a mechanism to avoid dysregulated T cell activation by pathobionts. Proc. Natl. Acad. Sci. USA 119:e2209624119. doi: 10.1073/pnas.2209624119, PMID: 36201539 PMC9565271

[ref167] Sassone-Corsi M. Nuccio S. P. Liu H. Hernandez D. Vu C. T. Takahashi A. A.. (2016). Microcins mediate competition among Enterobacteriaceae in the inflamed gut. Nature 540, 280–283. doi: 10.1038/nature20557, PMID: 27798599 PMC5145735

[ref168] Sato Y. Atarashi K. Plichta D. R. Arai Y. Sasajima S. Kearney S. M.. (2021). Novel bile acid biosynthetic pathways are enriched in the microbiome of centenarians. Nature 599, 458–464. doi: 10.1038/s41586-021-03832-5, PMID: 34325466

[ref169] Scarano A. Laddomada B. Blando F. De Santis S. Verna G. Chieppa M.. (2023). The chelating ability of plant polyphenols can affect Iron homeostasis and gut microbiota. Antioxidants (Basel) 12:630. doi: 10.3390/antiox12030630 36978878 PMC10045931

[ref170] Schuijt T. J. Lankelma J. M. Scicluna B. P. de Sousa F. Melo E. Roelofs J. J.. (2016). The gut microbiota plays a protective role in the host defence against pneumococcal pneumonia. Gut 65, 575–583. doi: 10.1136/gutjnl-2015-309728, PMID: 26511795 PMC4819612

[ref171] Schupack D. A. Mars R. A. T. Voelker D. H. Abeykoon J. P. Kashyap P. C. (2022). The promise of the gut microbiome as part of individualized treatment strategies. Nat. Rev. Gastroenterol. Hepatol. 19, 7–25. doi: 10.1038/s41575-021-00499-1, PMID: 34453142 PMC8712374

[ref172] Schurmann N. Forrer P. Casse O. Li J. Felmy B. Burgener A. V.. (2017). Myeloperoxidase targets oxidative host attacks to Salmonella and prevents collateral tissue damage. Nat. Microbiol. 2:16268. doi: 10.1038/nmicrobiol.2016.268, PMID: 28112722

[ref173] Schwartz D. J. Langdon A. E. Dantas G. (2020). Understanding the impact of antibiotic perturbation on the human microbiome. Genome Med. 12:82. doi: 10.1186/s13073-020-00782-x, PMID: 32988391 PMC7523053

[ref174] Schwerd T. Pandey S. Yang H. T. Bagola K. Jameson E. Jung J.. (2017). Impaired antibacterial autophagy links granulomatous intestinal inflammation in Niemann-pick disease type C1 and XIAP deficiency with NOD2 variants in Crohn's disease. Gut 66, 1060–1073. doi: 10.1136/gutjnl-2015-310382, PMID: 26953272 PMC5532464

[ref175] Sender R. Milo R. (2021). The distribution of cellular turnover in the human body. Nat. Med. 27, 45–48. doi: 10.1038/s41591-020-01182-9, PMID: 33432173

[ref176] Shandilya S. Kumar S. Kumar Jha N. Kumar Kesari K. Ruokolainen J. (2022). Interplay of gut microbiota and oxidative stress: perspective on neurodegeneration and neuroprotection. J. Adv. Res. 38, 223–244. doi: 10.1016/j.jare.2021.09.005, PMID: 35572407 PMC9091761

[ref177] Shi N. Li N. Duan X. Niu H. (2017). Interaction between the gut microbiome and mucosal immune system. Mil. Med. Res. 4:14. doi: 10.1186/s40779-017-0122-9, PMID: 28465831 PMC5408367

[ref178] Shin A. Preidis G. A. Shulman R. Kashyap P. C. (2019). The gut microbiome in adult and pediatric functional gastrointestinal disorders. Clin. Gastroenterol. Hepatol. 17, 256–274. doi: 10.1016/j.cgh.2018.08.054 30153517 PMC6314902

[ref179] Simpson B. W. Trent M. S. (2019). Pushing the envelope: LPS modifications and their consequences. Nat. Rev. Microbiol. 17, 403–416. doi: 10.1038/s41579-019-0201-x, PMID: 31142822 PMC6913091

[ref180] Singh A. K. Bishayee A. Pandey A. K. (2018). Targeting histone deacetylases with natural and synthetic agents: an emerging anticancer strategy. Nutrients 10:731. doi: 10.3390/nu10060731, PMID: 29882797 PMC6024317

[ref181] Singh T. P. Kadyan S. Devi H. Park G. Nagpal R. (2023). Gut microbiome as a therapeutic target for liver diseases. Life Sci. 322:121685. doi: 10.1016/j.lfs.2023.121685 37044173

[ref182] Srivastava M. Su D. Zhang H. Chen Z. Tang M. Nie L.. (2020). HMCES safeguards replication from oxidative stress and ensures error-free repair. EMBO Rep. 21:e49123. doi: 10.15252/embr.201949123, PMID: 32307824 PMC7271331

[ref183] Stephens M. von der Weid P. Y. (2020). Lipopolysaccharides modulate intestinal epithelial permeability and inflammation in a species-specific manner. Gut Microbes 11, 421–432. doi: 10.1080/19490976.2019.1629235, PMID: 31203717 PMC7524286

[ref184] Stevens J. Steinmeyer S. Bonfield M. Peterson L. Wang T. Gray J.. (2022). The balance between protective and pathogenic immune responses to pneumonia in the neonatal lung is enforced by gut microbiota. Sci. Transl. Med. 14:eabl3981. doi: 10.1126/scitranslmed.abl3981, PMID: 35704600 PMC10032669

[ref185] Stojanov S. Ravnikar M. Berlec A. Kreft S. (2021). Interaction between silver fir (*Abies alba*) wood water extract and lactobacilli. Pharmazie 76, 614–617. doi: 10.1691/ph.2021.1794, PMID: 34986959

[ref186] Strati F. Pujolassos M. Burrello C. Giuffre M. R. Lattanzi G. Caprioli F.. (2021). Antibiotic-associated dysbiosis affects the ability of the gut microbiota to control intestinal inflammation upon fecal microbiota transplantation in experimental colitis models. Microbiome 9:39. doi: 10.1186/s40168-020-00991-x, PMID: 33549144 PMC7868014

[ref187] Suez J. Zmora N. Zilberman-Schapira G. Mor U. Dori-Bachash M. Bashiardes S.. (2018). Post-antibiotic gut mucosal microbiome reconstitution is impaired by probiotics and improved by autologous FMT. Cell 174, 1406–1423.e16. doi: 10.1016/j.cell.2018.08.047, PMID: 30193113

[ref188] Sui H. Zhang L. Gu K. Chai N. Ji Q. Zhou L.. (2020). YYFZBJS ameliorates colorectal cancer progression in Apc(Min/+) mice by remodeling gut microbiota and inhibiting regulatory T-cell generation. Cell Commun. Signal 18:113. doi: 10.1186/s12964-020-00596-9, PMID: 32677955 PMC7367414

[ref189] Teng H. Wang Y. Sui X. Fan J. Li S. Lei X.. (2023). Gut microbiota-mediated nucleotide synthesis attenuates the response to neoadjuvant chemoradiotherapy in rectal cancer. Cancer Cell 41, 124–138.e6. doi: 10.1016/j.ccell.2022.11.013, PMID: 36563680

[ref190] Tilg H. Zmora N. Adolph T. E. Elinav E. (2020). The intestinal microbiota fuelling metabolic inflammation. Nat. Rev. Immunol. 20, 40–54. doi: 10.1038/s41577-019-0198-4, PMID: 31388093

[ref191] Tran T. T. T. Cousin F. J. Lynch D. B. Menon R. Brulc J. Brown J. R.. (2019). Prebiotic supplementation in frail older people affects specific gut microbiota taxa but not global diversity. Microbiome 7:39. doi: 10.1186/s40168-019-0654-1, PMID: 30867067 PMC6417215

[ref192] Tripathi A. Debelius J. Brenner D. A. Karin M. Loomba R. Schnabl B.. (2018). The gut-liver axis and the intersection with the microbiome. Nat. Rev. Gastroenterol. Hepatol. 15, 397–411. doi: 10.1038/s41575-018-0011-z, PMID: 29748586 PMC6319369

[ref193] Underhill D. M. Braun J. (2022). Fungal microbiome in inflammatory bowel disease: a critical assessment. J. Clin. Invest. 132:e155786. doi: 10.1172/JCI155786, PMID: 35229726 PMC8884899

[ref194] van der Post S. Birchenough G. M. H. Held J. M. (2021). NOX1-dependent redox signaling potentiates colonic stem cell proliferation to adapt to the intestinal microbiota by linking EGFR and TLR activation. Cell Rep. 35:108949. doi: 10.1016/j.celrep.2021.108949, PMID: 33826887 PMC10327654

[ref195] Vandeputte D. Falony G. Vieira-Silva S. Wang J. Sailer M. Theis S.. (2017). Prebiotic inulin-type fructans induce specific changes in the human gut microbiota. Gut 66, 1968–1974. doi: 10.1136/gutjnl-2016-313271, PMID: 28213610 PMC5739857

[ref196] Vona R. Pallotta L. Cappelletti M. Severi C. Matarrese P. (2021). The impact of oxidative stress in human pathology: focus on gastrointestinal disorders. Antioxidants (Basel) 10:201. doi: 10.3390/antiox10020201 33573222 PMC7910878

[ref197] Walsh B. J. C. Costa S. S. Edmonds K. A. Trinidad J. C. Issoglio F. M. Brito J. A.. (2022). Metabolic and structural insights into hydrogen sulfide Mis-regulation in *Enterococcus faecalis*. Antioxidants (Basel) 11:1607. doi: 10.3390/antiox11081607 36009332 PMC9405070

[ref198] Wang L. Cao Z. M. Zhang L. L. Li J. M. Lv W. L. (2022). The role of gut microbiota in some liver diseases: from an immunological perspective. Front. Immunol. 13:923599. doi: 10.3389/fimmu.2022.923599, PMID: 35911738 PMC9326173

[ref199] Wang Y. Chen C. Chen J. Sang T. Peng H. Lin X.. (2022b). Overexpression of NAG-1/GDF15 prevents hepatic steatosis through inhibiting oxidative stress-mediated dsDNA release and AIM2 inflammasome activation. Redox Biol. 52:102322. doi: 10.1016/j.redox.2022.102322, PMID: 35504134 PMC9079118

[ref200] Wang N. Guo Z. Zhang Y. Zhang P. Liu J. Cheng Y.. (2020). Effect on intestinal microbiota, bioaccumulation, and oxidative stress of *Carassius auratus* gibelio under waterborne cadmium exposure. Fish Physiol. Biochem. 46, 2299–2309. doi: 10.1007/s10695-020-00870-0, PMID: 32986141

[ref201] Wang Y. Kirpich I. Liu Y. Ma Z. Barve S. McClain C. J.. (2011). *Lactobacillus rhamnosus* GG treatment potentiates intestinal hypoxia-inducible factor, promotes intestinal integrity and ameliorates alcohol-induced liver injury. Am. J. Pathol. 179, 2866–2875. doi: 10.1016/j.ajpath.2011.08.039, PMID: 22093263 PMC3260853

[ref202] Wang Y. T. Liu T. Y. Shen C. H. Lin S. Y. Hung C. C. Hsu L. C.. (2022). K48/K63-linked polyubiquitination of ATG9A by TRAF6 E3 ligase regulates oxidative stress-induced autophagy. Cell Rep. 38:110354. doi: 10.1016/j.celrep.2022.110354, PMID: 35196483

[ref203] Wang Y. Zhang Z. Li B. He B. Li L. Nice E. C.. (2022a). New insights into the gut microbiota in neurodegenerative diseases from the perspective of redox homeostasis. Antioxidants (Basel) 11:2287. doi: 10.3390/antiox11112287 36421473 PMC9687622

[ref204] Wang Z. Zhao Y. (2018). Gut microbiota derived metabolites in cardiovascular health and disease. Protein Cell 9, 416–431. doi: 10.1007/s13238-018-0549-0, PMID: 29725935 PMC5960473

[ref205] Watanabe T. Kitani A. Murray P. J. Wakatsuki Y. Fuss I. J. Strober W. (2006). Nucleotide binding oligomerization domain 2 deficiency leads to dysregulated TLR2 signaling and induction of antigen-specific colitis. Immunity 25, 473–485. doi: 10.1016/j.immuni.2006.06.018 16949315

[ref206] Wieczorska K. Stolarek M. Stec R. (2020). The role of the gut microbiome in colorectal cancer: where are we? Where are we going? Clin. Colorectal Cancer 19, 5–12. doi: 10.1016/j.clcc.2019.07.006, PMID: 31678050

[ref207] Wilmes P. Martin-Gallausiaux C. Ostaszewski M. Aho V. T. E. Novikova P. V. Laczny C. C.. (2022). The gut microbiome molecular complex in human health and disease. Cell Host Microbe 30, 1201–1206. doi: 10.1016/j.chom.2022.08.016 36108612

[ref208] Wong S. H. Yu J. (2019). Gut microbiota in colorectal cancer: mechanisms of action and clinical applications. Nat. Rev. Gastroenterol. Hepatol. 16, 690–704. doi: 10.1038/s41575-019-0209-8, PMID: 31554963

[ref209] Woodby B. Penta K. Pecorelli A. Lila M. A. Valacchi G. (2020). Skin health from the inside out, Annu rev food. Sci. Technol. 11, 235–254. doi: 10.1146/annurev-food-032519-051722 31905017

[ref210] Wu Y. Yu X. Wang Y. Huang Y. Tang J. Gong S.. (2022). Ruscogenin alleviates LPS-triggered pulmonary endothelial barrier dysfunction through targeting NMMHC IIA to modulate TLR4 signaling. Acta Pharm. Sin. B 12, 1198–1212. doi: 10.1016/j.apsb.2021.09.017, PMID: 35530141 PMC9069402

[ref211] Xu S. Li X. Zhang S. Qi C. Zhang Z. Ma R.. (2023). Oxidative stress gene expression, DNA methylation, and gut microbiota interaction trigger Crohn's disease: a multi-omics Mendelian randomization study. BMC Med. 21:179. doi: 10.1186/s12916-023-02878-8, PMID: 37170220 PMC10173549

[ref212] Xue J. Ajuwon K. M. Fang R. (2020). Mechanistic insight into the gut microbiome and its interaction with host immunity and inflammation. Anim Nutr 6, 421–428. doi: 10.1016/j.aninu.2020.05.007, PMID: 33364458 PMC7750791

[ref213] Yardeni T. Tanes C. E. Bittinger K. Mattei L. M. Schaefer P. M. Singh L. N.. (2019). Host mitochondria influence gut microbiome diversity: a role for ROS. Sci. Signal. 12:eaaw3159. doi: 10.1126/scisignal.aaw3159, PMID: 31266851

[ref214] Yatsunenko T. Rey F. E. Manary M. J. Trehan I. Dominguez-Bello M. G. Contreras M.. (2012). Human gut microbiome viewed across age and geography. Nature 486, 222–227. doi: 10.1038/nature11053, PMID: 22699611 PMC3376388

[ref215] Yin J. Li Y. Han H. Chen S. Gao J. Liu G.. (2018). Melatonin reprogramming of gut microbiota improves lipid dysmetabolism in high-fat diet-fed mice. J. Pineal Res. 65:e12524. doi: 10.1111/jpi.12524, PMID: 30230594

[ref216] Yoon D. S. Lee K. M. Choi Y. Ko E. A. Lee N. H. Cho S.. (2022). TLR4 downregulation by the RNA-binding protein PUM1 alleviates cellular aging and osteoarthritis. Cell Death Differ. 29, 1364–1378. doi: 10.1038/s41418-021-00925-6, PMID: 35034101 PMC9287402

[ref217] Yuan Q. Tang B. Zhang C. (2022). Signaling pathways of chronic kidney diseases, implications for therapeutics. Signal Transduct. Target. Ther. 7:182. doi: 10.1038/s41392-022-01036-5, PMID: 35680856 PMC9184651

[ref218] Zhang S. Chen D. C. (2019). Facing a new challenge: the adverse effects of antibiotics on gut microbiota and host immunity. Chin. Med. J. 132, 1135–1138. doi: 10.1097/CM9.0000000000000245, PMID: 30973451 PMC6511407

[ref219] Zhang Y. Jelleschitz J. Grune T. Chen W. Zhao Y. Jia M.. (2022). Methionine restriction - association with redox homeostasis and implications on aging and diseases. Redox Biol. 57:102464. doi: 10.1016/j.redox.2022.102464, PMID: 36152485 PMC9508608

[ref220] Zhang B. Zeng M. Wang Y. Li M. Wu Y. Xu R.. (2022). Oleic acid alleviates LPS-induced acute kidney injury by restraining inflammation and oxidative stress via the Ras/MAPKs/PPAR-gamma signaling pathway. Phytomedicine 94:153818. doi: 10.1016/j.phymed.2021.153818 34798521

[ref221] Zhao L. Zhang F. Ding X. Wu G. Lam Y. Y. Wang X.. (2018). Gut bacteria selectively promoted by dietary fibers alleviate type 2 diabetes. Science 359, 1151–1156. doi: 10.1126/science.aao5774, PMID: 29590046

[ref222] Zhou Y. Hu L. Tang W. Li D. Ma L. Liu H.. (2021). Hepatic NOD2 promotes hepatocarcinogenesis via a RIP2-mediated proinflammatory response and a novel nuclear autophagy-mediated DNA damage mechanism. J. Hematol. Oncol. 14:9. doi: 10.1186/s13045-020-01028-4, PMID: 33413510 PMC7791875

[ref223] Zhou J. Li M. Chen Q. Li X. Chen L. Dong Z.. (2022). Programmable probiotics modulate inflammation and gut microbiota for inflammatory bowel disease treatment after effective oral delivery. Nat. Commun. 13:3432. doi: 10.1038/s41467-022-31171-0, PMID: 35701435 PMC9198027

[ref224] Ziemons J. Smidt M. L. Damink S. O. Rensen S. S. (2021). Gut microbiota and metabolic aspects of cancer cachexia. Best Pract. Res. Clin. Endocrinol. Metab. 35:101508. doi: 10.1016/j.beem.2021.101508, PMID: 33648847

[ref225] Zmora N. Suez J. Elinav E. (2019). You are what you eat: diet, health and the gut microbiota. Nat. Rev. Gastroenterol. Hepatol. 16, 35–56. doi: 10.1038/s41575-018-0061-2, PMID: 30262901

